# Chirality Effects in Peptide‐Based Dynamic Combinatorial Chemistry

**DOI:** 10.1002/chem.202501298

**Published:** 2025-05-19

**Authors:** Alice Gable, Emmi Pohjolainen, Gerrit Groenhof, Fabien B. L. Cougnon

**Affiliations:** ^1^ Department of Chemistry, Nanoscience Center University of Jyväskylä Survontie 9 C Jyväskylä 40014 Finland

**Keywords:** chirality, cysteine‐rich peptides, dynamic combinatorial chemistry, macrocycles, supramolecular

## Abstract

Naturally occurring peptides are almost exclusively composed of L‐amino acids, and the incorporation of D‐amino acids can profoundly alter their ability to fold and self‐assemble. Here we explore the effects of chirality on the formation of disulfide dynamic combinatorial libraries (DCLs) generated by short cysteine‐rich peptides. Our findings consistently show that heterochiral tripeptides form more diverse DCLs than their homochiral counterparts. The most complex library appears to encompass all possible cyclic species up to 19mers. Given that each of these species exists as a mixture of parallel and antiparallel isomers, we estimate this library to contain a total of 2,045 distinct compounds—a remarkable result considering that the library generated by the analogous homochiral peptide predominantly contains two dimers. In certain situations, peptide chirality also affects the relative stability of parallel and antiparallel isomers. Taken together, these results show that small changes in peptide chirality can be dramatically amplified through the formation of cyclic species.

## Graphical abstract

Heterochiral peptides are known to exhibit unique folding and self‐assembly behaviors. Yet, the impact of heterochirality in peptide‐based dynamic combinatorial chemistry remains largely unexplored. This study reveals that chirality effects can be used to increase diversity in dynamic combinatorial libraries, significantly enhancing the potential applications of these systems in the discovery of new drugs, biosensors, and adaptive materials.

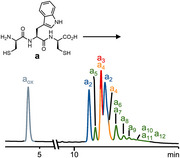

## 1 Introduction

Homochirality is a defining feature of biomolecules. In living organisms, nearly all proteins and peptides are composed of L‐amino acids. D‐amino acids are rare and primarily found in bacterial cell walls, where they are introduced through post‐translational modifications.^[^ ^1^, ^2^, ^3^, ^4^, ^5^ ^]^ This rarity has given D‐amino acids a unique advantage in biotechnologies.^[^ ^6^, ^7^, ^8^, ^9^, ^10^, ^11^, ^12^ ^]^ Peptides exclusively composed of D‐amino acids, for example, are excellent candidates for the treatments of various diseases because they are not recognized by endogenous proteases. The design of heterochiral peptides, composed of both L‐ and D‐amino acids, has garnered increased attention in recent years. Combining mirror‐image amino acids allows to expand the available conformational space of peptides by sampling more diverse backbone dihedral angles φ and ψ. As a result, heterochirality can endow peptides with unusual folding properties^[^ ^13^, ^14^ ^]^ and distinct self‐assembly behaviors.^[^ ^15^, ^16^, ^17^, ^18^, ^19^, ^20^, ^21^, ^22^ ^]^ Examples of natural heterochiral peptides include the membrane pore‐forming gramicidin^[^ ^23^ ^]^ and polytheonamide.^[^ ^24^ ^]^ These peptides consist of amino acids with alternating chirality, enabling the formation of β‐helical conformations essential to their biological activity. Even small peptides exhibit pronounced chirality effects, which can be transmitted across length scales through their assembly into helical nanofibers with different handedness^[^ ^25^, ^26^ ^]^ and gels.^[^ ^27^, ^28^, ^29^, ^30^ ^]^

The difference in conformational behavior between homo‐ and heterochiral peptides could prove particularly relevant in the context of dynamic combinatorial chemistry.^[^ ^31^, ^32^, ^33^, ^34^, ^35^, ^36^ ^]^ Dynamic combinatorial chemistry is concerned with the creation of thermodynamically controlled chemical libraries that can adapt in response to external stimuli. Dynamic combinatorial libraries (DCLs) are generated by connecting simple molecular building blocks through reversible covalent bonds, allowing the system to continuously equilibrate and respond to changes in conditions.^[^ ^37^, ^38^, ^39^, ^40^, ^41^, ^42^, ^43^, ^44^ ^]^ For instance, the introduction of a target biomolecule can cause a reorganization of the mixture based on the affinity of library components for the biomolecule.^[^ ^37^, ^38^, ^39^, ^40^ ^]^ This feature has been exploited to identify highly specific molecular receptors and novel catalysts.^[^ ^41^, ^42^, ^43^, ^44^, ^45^, ^46^ ^]^ Complex molecular architectures, such as interlocked molecules^[^ ^47^, ^48^, ^49^ ^]^ and unusual foldamers,^[^ ^50^, ^51^, ^52^, ^53^, ^54^ ^]^ have also been shown to emerge spontaneously in DCLs.

Most DCLs are created from synthetic building blocks, which may be functionalized with amino acids or other natural synthons.^[^ ^41^, ^43^, ^50^, ^52^, ^53^, ^54^, ^55^, ^56^, ^57^ ^]^ Pure peptides can also serve as building blocks, as the naturally occurring amino acid cysteine can oxidize in aerated aqueous solutions to form disulfide bonds that are reversible under physiological pH. However, the composition of DCLs generated from homochiral peptides tend to be relatively simple and dominated by the smallest available species, such as closed monomers and dimers.^[^ ^58^, ^59^, ^60^ ^]^ Expanding the diversity of peptide‐based DCLs would significantly enhance their applicability in the discovery of new drugs, biosensors, and adaptative materials.

In this article, we explore the influence of chirality in aqueous DCLs generated by short cysteine‐rich peptides. We demonstrate that heterochiral tripeptides can form exceptionally complex libraries, featuring cyclic species of unprecedented size (up to 19mers!) for this type of peptide‐based dynamic combinatorial systems. We also show that the chirality of these peptides can bias the equilibria in favor of specific constitutional isomers (e.g., antiparallel over parallel dimers) in specific cases.

## 2 Results and Discussion

Homo‐ and heterochiral peptides were synthesized using solid‐phase peptide synthesis and purified by preparative reverse‐phase high performance liquid chromatography (HPLC). DCLs were generated by solubilizing these peptides at specific concentrations in ammonium bicarbonate buffer (50 mM, pH 8). The libraries were stirred under air for two days before being analyzed by tandem reverse‐phase ultra‐high performance liquid chromatography–mass spectrometry (UHPLC‐MS). This reaction time was selected based on the observation that the libraries reached thermodynamic equilibrium within approximately 48 hours (Figure S7), while extending the reaction time beyond three days led to the unwanted apparition of overoxidized sulfoxide‐ and sulfone‐type products (Figure S8).

### 2.1 DCLs Generated From LLL‐homochiral Tripeptides

We first investigated the behavior of LLL‐homochiral tripeptides with the sequence CXC. In this design, the terminal cysteines (C) provide the thiols necessary to the formation of disulfide DCLs. Amino acid X represents either isoleucine I (**1a**), leucine L (**2a**), tyrosine Y (**3a**), or phenylalanine F (**4a**). We exclusively chose hydrophobic amino acids at position X, anticipating that this feature would promote hydrophobically driven assembly and folding.

**Figure 1:**
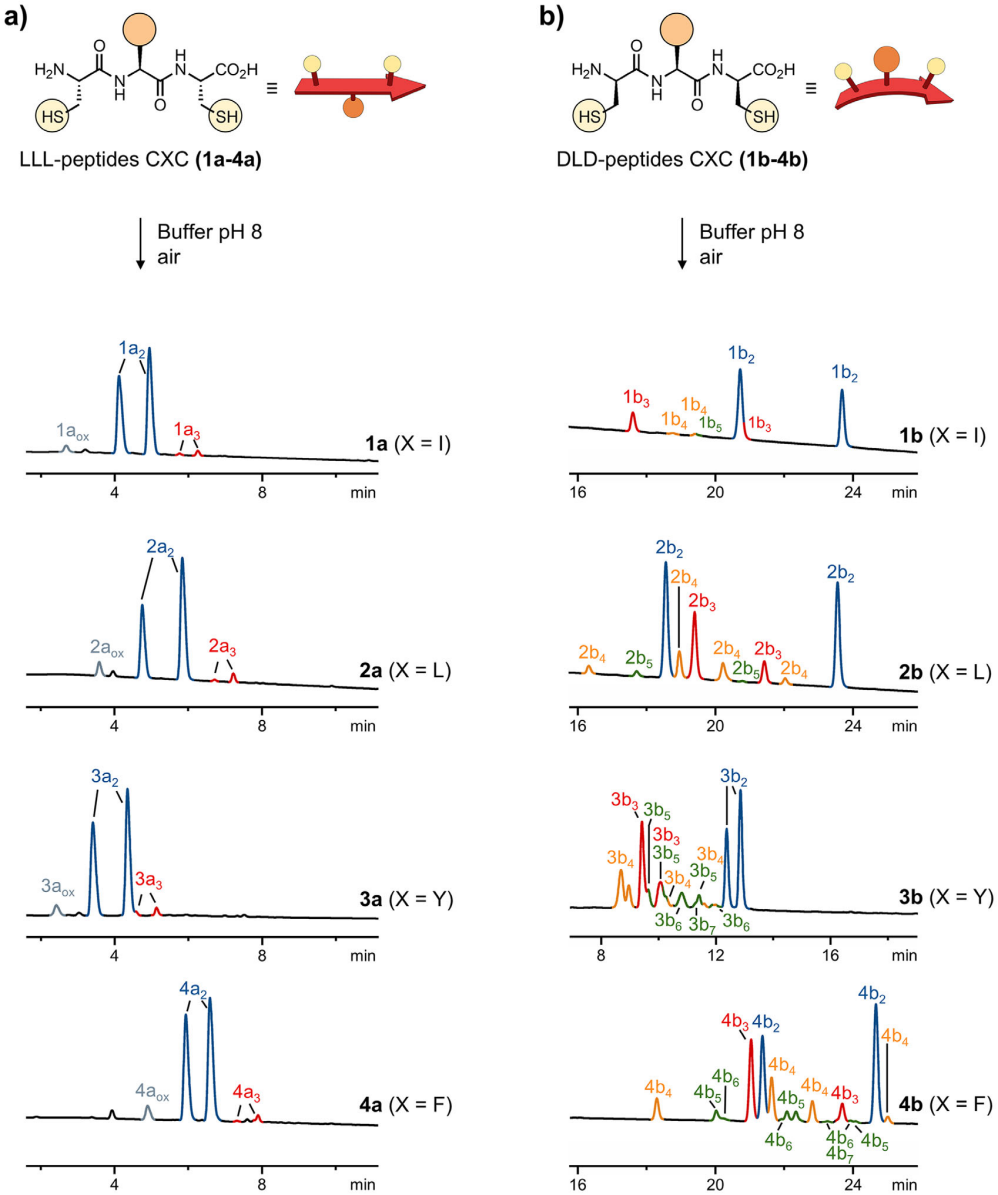
Chromatograms of DCLs generated by incubating (a) LLL‐homochiral and (b) DLD‐heterochiral tripeptides (10 mM) with the sequence CXC in ammonium bicarbonate buffer pH 8 for 48 hours. Here, X = isoleucine I (**1**), leucine L (**2**), tyrosine Y (**3**), or phenylalanine F (**4**). Elution conditions for (a): C18 XBridge BEH column with a linear gradient from 5% to 80% acetonitrile (0.1% trifluoroacetic acid) in water over 30 minutes. Elution conditions for (b): C18 XSelect peptide CSH with a linear gradient from 15% to 40% acetonitrile (0.1% trifluoroacetic acid) in water over 30 minutes. Absorbance was recorded at 220 nm. Note: all the DLD‐heterochiral tripeptides form appreciable amounts of closed monomers, which are not shown in (b) because they elute much earlier than the other macrocycles. Full chromatograms for both LLL‐homochiral and DLD‐heterochiral libraries, recorded under identical elution conditions for direct comparison, are provided in the Supporting Information (Figures S14‐S20).

In line with previous reports,^[^ ^58^, ^60^ ^]^ homochiral peptides **1a**‐**4a** (10 mM) generated simple libraries predominantly composed of parallel and antiparallel dimers (Figure 1a). Both dimers were formed in nearly equal proportions, and only trace amounts of closed monomer and trimers were observed. This composition was largely independent of the nature of the central amino acid X, and the products consistently eluted based on their size: first the monomer, followed by the dimers, and finally the trimers.

The composition of the libraries is determined by the thermodynamic stability of these macrocycles. Yet, the prevalent formation of dimers may be rationalized using a simplified model. In their relaxed state, homochiral peptides composed of amino acids with β‐sheet propensity typically adopt a conformation in which the three side chains alternate on opposite sides of the backbone, as illustrated in the cartoon representation in Figure 1a. In our case, the two terminal thiols lie on the same side of the backbone, a configuration that is favorable to the formation of dimers. The central amino acid side chains cause no steric interference, as they lie on the opposite side of the backbone.

The resulting macrocycles display only a few hydrophobic side chains and have limited opportunities for intramolecular hydrogen bonding. These features are not conducive to the formation of well‐defined secondary structures. The absence of stabilizing interactions was confirmed through concentration‐dependent experiments. In the presence of stabilizing interactions, dimers and trimers would persist even at lower concentrations. However, preparing the libraries in dilute conditions (e.g., 0.1 mM or 1 mM peptide) simply resulted in an increase in the proportion of closed monomer at the expense of the dimers and trimers (Figures S9‐S12). Moreover, the libraries composition was not affected by the addition of high concentrations of inorganic salt (up to 1 M NaCl, Figure S13), which increases the medium ionic strength and promotes the amplification of folded species–provided such species can form in the system.

### 2.2 DCLs Generated From DLD‐heterochiral Tripeptides

To our delight, the behavior of the analogous DLD‐heterochiral tripeptides (**1b**‐**4b**, Figure 1b) proved to be markedly different from that of their homochiral counterparts.

These peptides (10 mM) produced libraries composed of a broader range of products, including higher oligomers such as 4mers, 5mers, and beyond. Moreover, a closer examination of the library compositions revealed a significant influence of the side chain attached to the central amino acid. When this side chain was purely aliphatic, as in the cases of isoleucine (**1b**) and leucine (**2b**), these libraries remained relatively simple. Peptide **1b** produced the simplest library, consisting of closed monomer (57%, not shown in Figure 1b), dimers (35%), trimers (6%), and traces of 4mers and 5mers (< 2% total). These percentages were estimated by integration of the UHPLC peak areas. Peptide **2b** produced a comparable library, albeit with a higher proportion of trimers (12%), 4mers, and 5mers (9% total). When the side chain was aromatic, as in the cases of tyrosine (**3b**) and phenylalanine (**4b**), the libraries were significantly more complex and contained greater amounts of trimers (> 20%) and higher‐order cyclic oligomers, including 6mers and 7mers (> 18%). Increasing peptide concentration to 25 mM even led to the formation of larger species, such as 8mers and 9mers (Figure S19). Integration of the peaks corresponding to the dimers, trimers and tetramers revealed that these species were formed as a statistical mixture of isomers (see Table S1 for the calculation of the number of possible isomers for each *n*‐mer). These relative ratios could not be determined for larger *n*‐mers due to their lower abundance and the overlap of the corresponding peaks, but it is likely that these species also form as statistical mixtures.

In general, the complexity of the libraries correlated with the size and hydrophobicity of the central amino acid side chain, as well as its ability to participate in aromatic π‐π stacking. Here again, this result may be rationalized using the simplified model introduced earlier. In the DLD‐heterochiral peptide, the three side chains lie on the same side of the backbone, as showed in the cartoon in Figure 1b. This configuration causes all the side chains to converge toward the center of the *n*‐mers. Since larger side chains need more space for packing, steric constraints promote the formation larger species.

Small macrocycles (e.g., monomer, dimers, and trimers) can still form but they are likely too simple to adopt well‐defined folded states. On the other hand, the larger *n*‐mers are sufficiently complex to form stable secondary structures that allow them to efficiently minimize the hydrophobic surface area exposure to water. This conclusion is supported by the seemingly random retention times of the heterochiral *n*‐mers in the chromatograms presented in Figure 1b. Indeed, folding can significantly alter the surface of interaction between the peptides and the solid phase employed in chromatography, leading to unpredictable variations in retention times.

The amplification of the higher‐order oligomers derived from peptide **3b** (10 mM) upon addition of 1 M NaCl (Figure S23) confirmed their ability to minimize their hydrophobic surface area via folding. Unfortunately, the addition of salt had no effect on the library produced from peptide **2b** and led to precipitation with peptides **1b** and **4b**, preventing us from drawing any conclusions in these cases.

Concentration‐dependent studies (Figures S15‐S21) highlighted the greater stability of the large heterochiral *n*‐mers compared to the large homochiral *n*‐mers. The library derived from peptide **4b**, for example, still produced 4mers and 5mers at 1 mM. Trimers even remain detectable when the library was prepared at concentrations as low as 0.1 mM. For comparison, homochiral trimers were only observed at a concentration of 10 mM and were absent in more dilute conditions.

Finally, mixtures of homo‐ and heterochiral tripeptides only resulted in statistical combinations, with no preferential formation of specific products. Representative examples of these experiments are shown in Figures S24‐S26. The absence of self‐sorting may be explained by the observation that mixing different peptides predominantly led to the formation of small *n*‐mers (monomers and dimers). Selectivity may have been observed if larger proportions of larger *n*‐mers had formed.

### 2.3 DCLs Generated from LLL‐, DLD‐, and LLD‐tripeptides with Sequence CWC

The contrasting behaviors of LLL‐homochiral and DLD‐heterochiral peptides led us to question how LLD‐heterochiral peptides would behave. Since chirality effects are more pronounced when the tripeptide features a central amino acid with a large aromatic side chain, we selected tripeptide CWC (where W is tryptophan) as a suitable candidate to answer this question.

**Figure 2:**
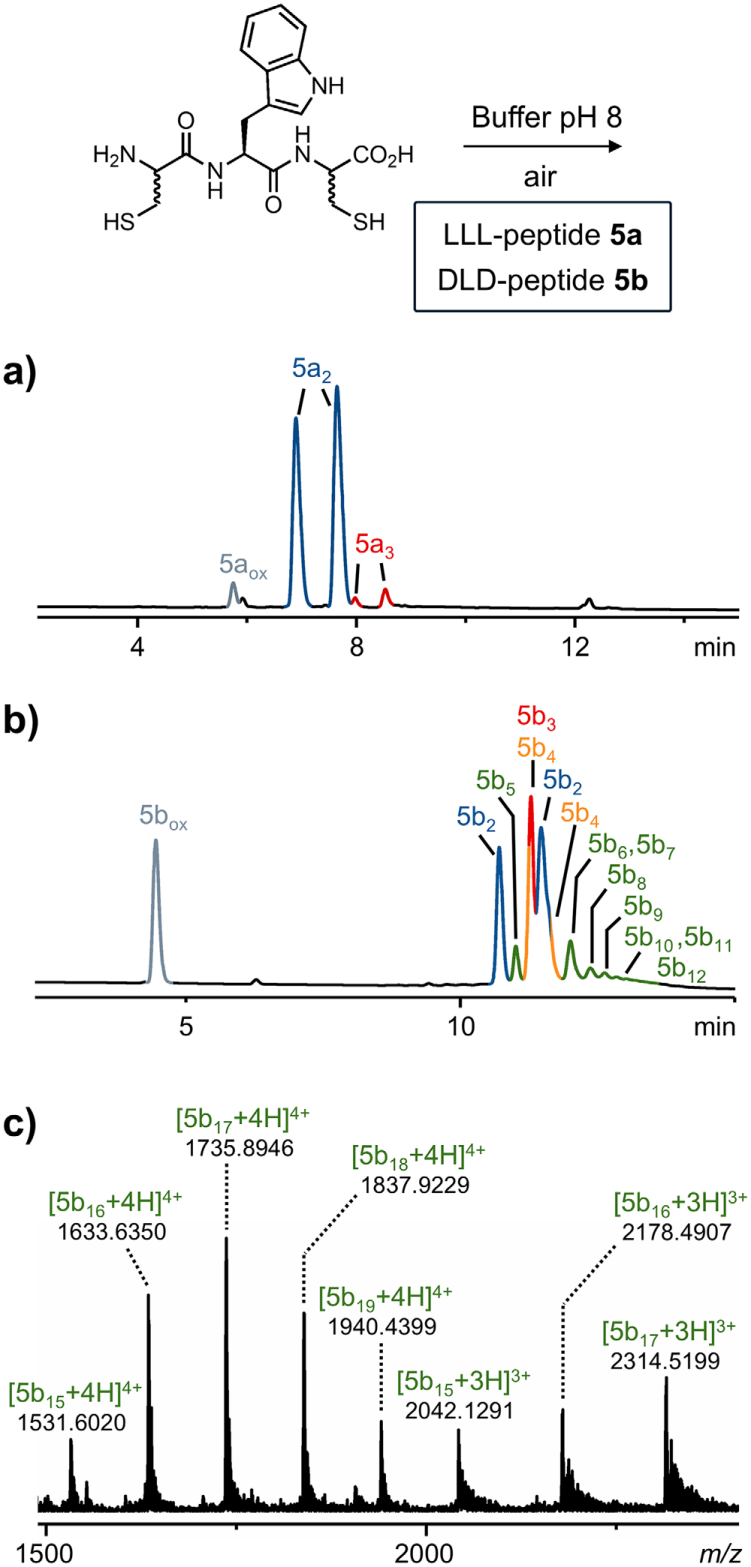
Chromatograms of the DCLs generated by incubating (a) LLL‐homochiral (**5a**) and (b) DLD‐heterochiral (**5b**) tripeptides CWC (10 mM) in ammonium bicarbonate buffer pH 8 for 48 hours. Elution conditions: C18 XBridge BEH column with a linear gradient from 5% to 80% acetonitrile (0.1% trifluoroacetic acid) in water over 30 minutes. (c) ESI(+)‐MS spectrum of the higher oligomers observed when the library was generated from higher concentration (25 mM) of DLD‐heterochiral tripeptide CWC (**5b**).

Three versions of this new tripeptide were then synthesized: LLL‐homochiral (**5a**), DLD‐heterochiral (**5b**), and LLD‐heterochiral (**5c**). Without surprise, LLL‐homochiral peptide **5a** and DLD‐heterochiral peptide **5b** followed the trends previously established. Peptide **5a** formed a simple library (Figure 2a), primarily consisting of two dimers present in a 1:1 statistical ratio. In contrast, peptide **5b** produced a highly complex library (Figure 2b), encompassing all the macrocyclic oligomers from the monomer up to 12mers. At a higher concentration (25 mM), the library of peptide **5b** even produced trace amounts of 19mers (Figures 2c and S31). Consistent with our previous findings, these *n*‐mers were formed as statistical mixtures of all possible isomers. By calculating the number of isomers for each *n*‐mer (Table S1), we estimate these libraries contain between 189 components (at 10 mM) and 2045 components (at 25 mM). To the best of our knowledge, such diversity has never been observed before in DCLs generated by a single peptide.

**Figure 3:**
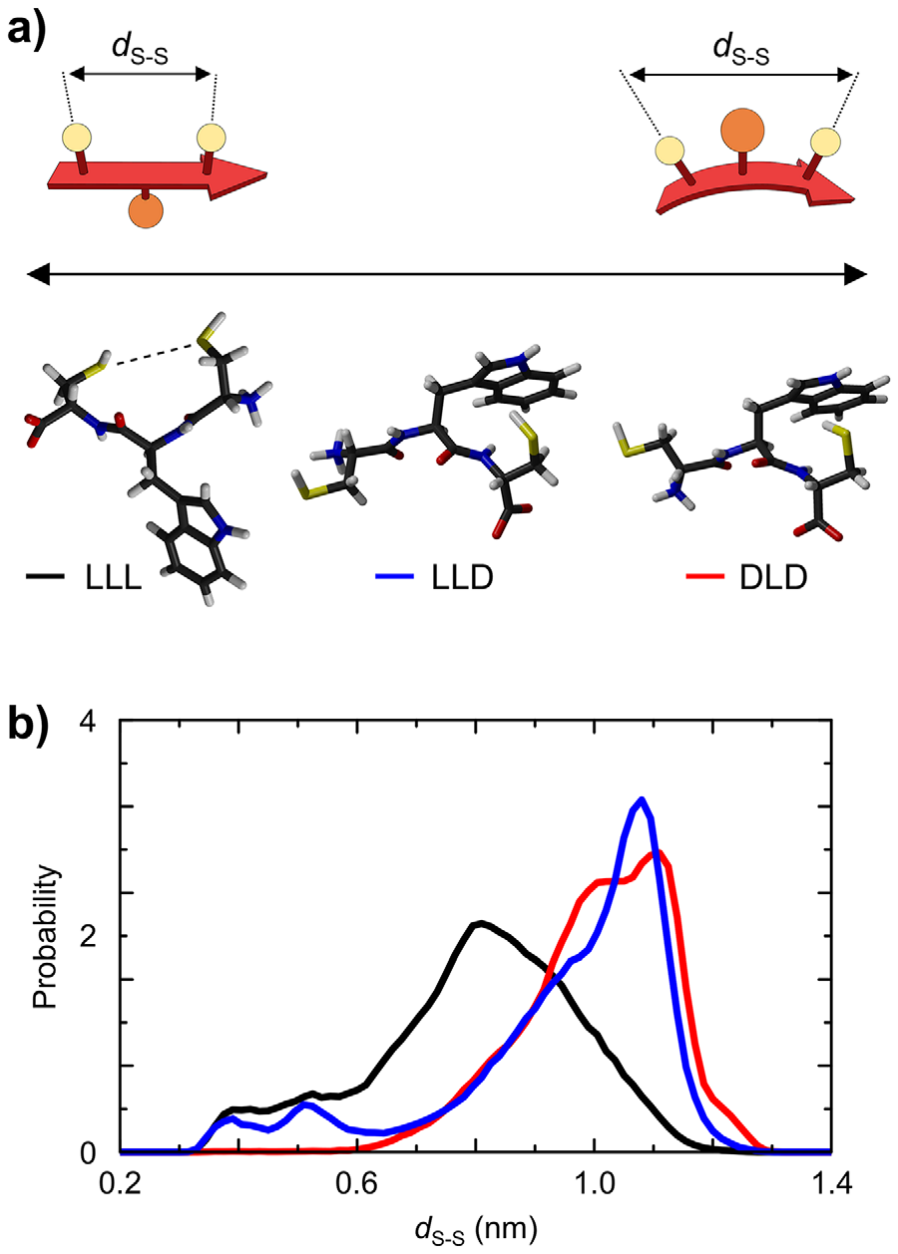
(a) Representative structure and (b) distribution of the sulfur –sulfur distance of the LLL‐homochiral (**5a**), DLD‐heterochiral (**5b**), and LLD‐heterochiral (**5c**) tripeptides CWC in MD simulations.

Molecular dynamics (MD) simulations were conducted on the LLL‐homochiral (**5a**) and DLD‐heterochiral (**5b**) tripeptides to better understand their conformational behavior and explain the differences in diversity between the corresponding libraries. The simulations (Figure 3) confirm that the three side chains of peptide **5a** alternate on opposite sides of the backbone, while those of peptide **5b** are positioned on the same side. The latter arrangement introduces steric hindrance and affects backbone curvature,^[^ ^29^ ^]^ as illustrated in the cartoon representations. We used the distance between the sulfur atoms of the two terminal cysteines *d*_S‐S_ as a marker to quantify this conformational effect. The average distance between sulfur atoms proved to be significantly larger in the DLD‐heterochiral peptide (1.01 ± 0.12 nm) than in the LLL‐homochiral (0.77 ± 0.19 nm), supporting the model used to explain the tendency of DLD‐heterochiral peptides to form larger macrocycles.

**Figure 4:**
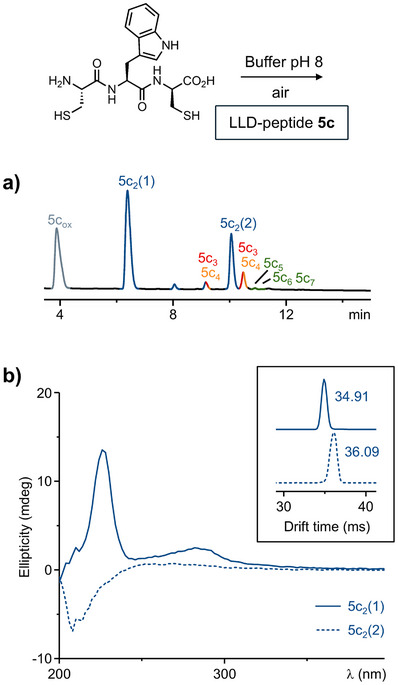
(a) Chromatogram of the DCLs generated by incubating LLD‐heterochiral tripeptide CWC (**5c**, 10 mM) in ammonium bicarbonate buffer pH 8 for 48 hours. Elution conditions: C18 XBridge BEH column with a linear gradient from 5% to 80% acetonitrile (0.1% trifluoroacetic acid) in water over 30 minutes. (b) CD spectrum of the corresponding monomers and dimers. Insert: Representation of the ion‐mobility mass spectrometry drift times at *m/z* 839, corresponding to the ion [**5c**_2_+Na]^+^.

The average distance between the sulfurs was intermediate in LLD‐heterochiral peptide **5c** (0.90 ± 0.20 nm). As a result, peptide **5c** (Figure 4a) exhibited an intermediate behavior: it formed larger oligomers compared to **5a**, but in fewer quantities and with less variety than **5b**. This time, however, we noted a clear discrimination between the two isomeric dimers, with a 2:1 relative ratio between the first and second eluting dimers. Such discrimination had not been observed in any of the samples analyzed up to that point. The same discrimination may also apply to the higher‐order oligomers, but their low abundance prevented us to confirm this hypothesis.

The two dimers **5c**_2_ were isolated by preparative reverse‐phase HPLC for further characterization by circular dichroism (CD) spectroscopy. Despite their small size and limited structural complexity, they displayed strikingly different CD signatures between 200 nm and 400 nm (Figure 4b). The first dimer displayed positive peaks between 250 nm to 310 nm (corresponding to electronic transitions of the aromatic indole side chain of tryptophan) and at 220 nm (corresponding to electronic transitions of the backbone peptide bonds). These CD features, along with the higher yield of formation of this first dimer, suggest it can adopt a relatively well‐defined secondary structure. The second dimer, which was formed in a lower proportion, exhibited a much weaker CD signature with a negative peak around 210 nm typically observed with peptides lacking a dominant secondary structure (i.e., random coil). Similar weak CD signatures were obtained for the dimers generated from either **5a** or **5b**, which were also isolated by preparative HPLC. These data are provided in the supplementary information (Figures S35‐S37).

Ion‐mobility spectroscopy‐mass spectrometry (IM‐MS, insert of Figure 4b and Table S2) corroborated the conclusions drawn from CD spectroscopy. The most notable difference in drift times and collision cross section (CCS) values was obtained between the two dimers **5c**_2_. The drift time of the first dimer, which appeared to be better folded, was shorter than that of the second dimer (34.91 ms vs. 36.09 ms), and the CCS values followed the same trend (^DT^CCS_He_ = 259.9 Å^2^ for the first dimer vs. 268.8 Å^2^ for the second dimer). These differences are less pronounced with dimers **5a**_2_ and **5b**_2_.

**Figure 5:**
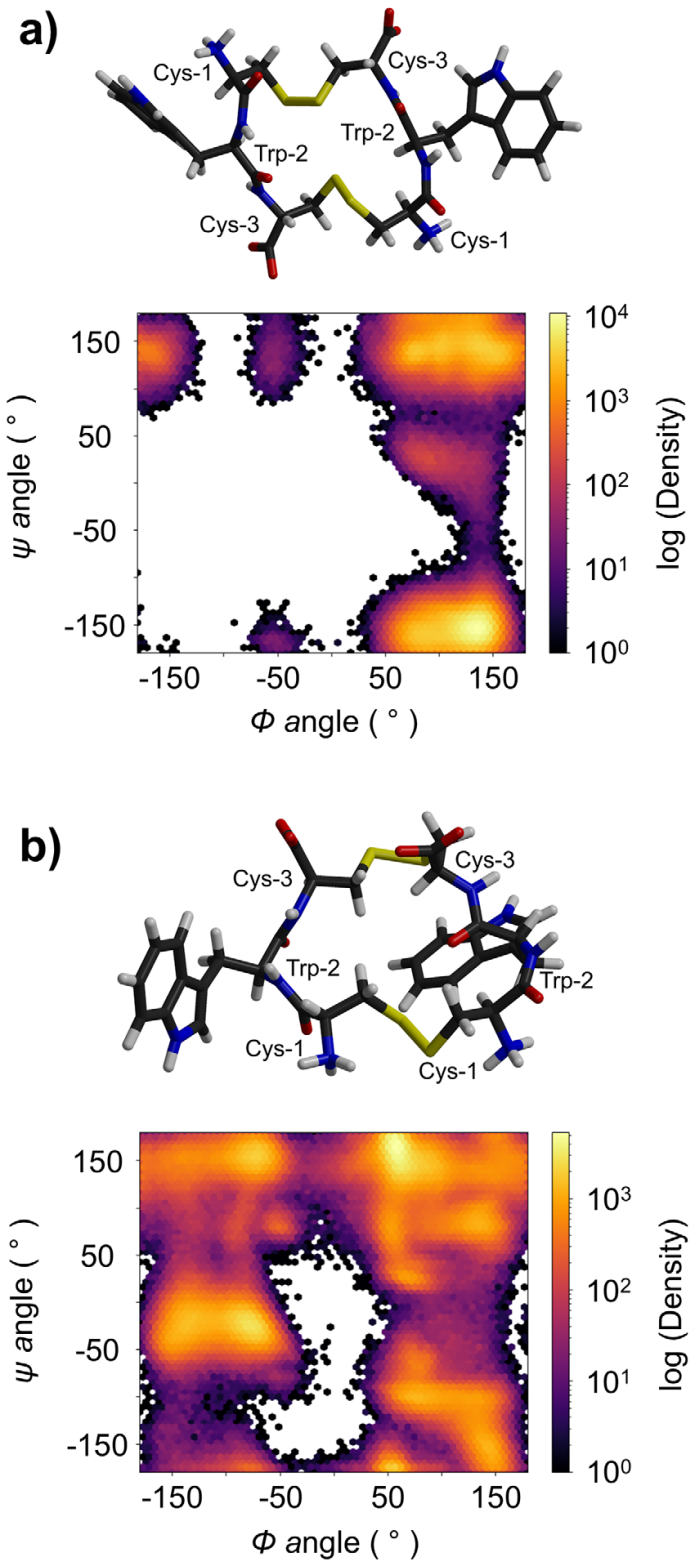
Representative structure and Ramachandran plots of (a) antiparallel and (b) parallel dimers **5c**_2_ obtained from cluster analysis of MD trajectories. The Ramachandran plots suggest that the antiparallel dimer is significantly more structured than the parallel dimer.

Ramachandran plots obtained from MD trajectories of antiparallel and parallel dimers **5c**_2_ in water (Figure 5a) suggest that the predominant dimer is the antiparallel dimer. Indeed, the antiparallel dimer exhibits a well‐defined secondary structure, as indicated by a more restricted sampling of the backbone torsions. In contrast, for all other dimers (parallel **5c**_2_, as well as **5a**_2_ and **5b**_2_), the backbone sampled the entire dihedral space during the simulation (Figures 5b and S39‐40).

### 2.4 DCLs Generated from Tetrapeptides

The results obtained with tripeptides prompted us to explore the role of chirality in DCLs derived from longer peptides featuring an extended CXXC sequence. Given that increasing the length of the central hydrophobic region inevitably reduces peptide solubility, we first sought to test the validity of this approach by extending the sequence of the CIC peptide, which had exhibited the highest solubility in our earlier series.

We synthesized the corresponding CIIC tetrapeptides with the following chirality: LLLL‐homochiral (**6a**), DLDL‐heterochiral (**6b**), and DDLL‐heterochiral (**6c**). As anticipated, the solubility of these tetrapeptides was significantly lower than that of the tripeptides, and libraries could not be produced with peptide concentrations exceeding 5 mM. Even at the highest achievable concentration of 5 mM, the oxidation of these tetrapeptides yielded only closed monomers (Figure S41). This result is likely due to their increased flexibility, which promotes the formation of intramolecular disulfide bonds. Attempts to alter the conditions by adding inorganic salt or mixing different tetrapeptides had no effect on this outcome. Consequently, the role of chirality in tetrapeptide was not explored further.

## 3 Conclusion

In conclusion, we demonstrated that the chirality of amphiphilic tripeptides CXC has a significant impact on the complexity and diversity of the corresponding DCLs. Homochiral LLL‐tripeptides formed simple libraries dominated by parallel and antiparallel dimers, whereas heterochiral DLD‐tripeptides generated more complex libraries containing higher‐order oligomers. The largest *n*‐mers were formed when bulky aromatic side chains were appended to the central amino acid X.

The libraries generated by tripeptides CWC best illustrate the difference in behavior between homo‐ and heterochiral peptides. In this case, the LLL‐peptide only produced dimers, and the DLD‐peptide yielded every possible oligomer up to 19mers. The LLD‐peptide generated a library of intermediate complexity but allowed for an unprecedented discrimination between parallel and antiparallel isomers, with one dimer being twice as stable as the other one. The intense CD signature and shorter drift time of the most stable dimer suggest that it adopts a well‐defined folded state, unlike the other homo‐ or heterochiral dimers.

These chirality effects were observed only with tripeptides, and the longer CXXC tetrapeptides yielded oxidized closed monomers, regardless of their chirality. The observation that the simplest libraries reported in this study contains a single component (i.e., a closed monomer), while the most complex library may include up to 2045 components (i.e., all the possible species up to 19mers), underscores the complexity of chirality effects and highlights how little we understand about the conformational behavior of simple peptides composed of just three or four amino acids. Developing a better appreciation of chirality effects through studies like this one may ultimately enable access to entirely new types of protein folds.

## Supporting Information

The authors have cited additional references within the Supporting Information.^[^ ^61^, ^62^, ^63^, ^64^, ^65^, ^66^, ^67^, ^68^, ^69^, ^70^, ^71^ ^]^

## Conflict of Interests

There are no conflicts to declare.

## Supporting information



Supporting Information

## Data Availability

The data that support the findings of this study are available from the corresponding author upon reasonable request.
