# Supplementary material for: Chirality Effects in Peptide‐Based Dynamic Combinatorial Chemistry
Source: Chemistry. 2025 May 19;31(35):e202501298. doi: 10.1002/chem.202501298 (PMC12188161; doi:10.1002/chem.202501298)
Supplement: Supplementary file 1 — Supporting Information [file CHEM-31-e202501298-s001.pdf]

Supporting Information for:

Chirality Effects in Peptide-Based Dynamic Combinatorial Chemistry

Alice Gable[a], Emmi Pohjolainen[a], Gerrit Groenhof[a] and Fabien B. L. Cougnon[a]*

[a] Department of Chemistry, Nanoscience Center, University of Jyväskylä, P.O. Box 35, FI-40014 JYU, Finland

Corresponding Author
* fabien.b.l.cougnon@jyu.fi

n-mer	Number of isomers for each *n*-mer	Cumulated number of isomers from 1mer to *n*-mer
1mer	1	1
2mer	2	3
3mer	2	5
4mer	4	9
5mer	4	13
6mer	8	21
7mer	8	29
8mer	16	45
9mer	16	61
10mer	32	93
11mer	32	125
12mer	64	189
13mer	64	253
14mer	128	381
15mer	128	509
16mer	256	765
17mer	256	1021
18mer	512	1533
19mer	512	2045

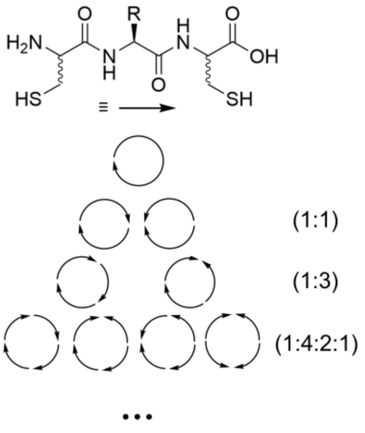

Table S1. Number of head-to-head and head-to-tail isomers for every *n*-mer formed in peptide-based dynamic combinatorial libraries.

Table of Contents

1. Methods.. S5

2. Solid-Phase Peptide Synthesis (SPPS)... S7

   2.1 Peptides 1 .... S8
   2.2 Peptides 2 .... S9
   2.3 Peptides 3 .... S10
   2.4 Peptides 4 .... S11
   2.5 Peptides 5 .... S12
   2.6 Peptides 6 .... S14

3. Reversibility test and reaction time optimization S16

4. Libraries generated from homochiral LLL-tripeptides S18

   4.1 Concentration-dependent libraries S18
   4.2 Effect of High salt concentration (1M NaCl) S22

5. Libraries generated from heterochiral DLD-tripeptides S23

   5.1 Comparison of libraries generated from homochiral LLL- and heterochiral DLD-tripeptides and concentration-dependent libraries.................................. S23

      5.1.1 Peptide 1b...S23
      5.1.2 Peptide 2b...S25
      5.1.3 Peptide 3b... S27
      5.1.4 Peptide 4b... S29

   5.2 Effect of High salt concentration (1M NaCl) S32

6. Libraries generated from mixture of tripeptides S33

   6.1 Libraries generated from mixture of different homochiral LLL-tripeptides S33
   6.2 Libraries generated from mixture of different heterochiral DLD- tripeptides.. S34
   6.3 Libraries generated from mixture of homochiral LLL- and heterochiral DLD-tripeptides analogues S36

7. Dynamic combinatorial libraries generated from tripeptides 5a-c S37

   7.1 Concentration-dependent libraries of peptide 5a S37
   7.2 Libraries generated from peptides 5a and 5b at 10 Mm S38
   7.3 Concentration-dependent libraries of peptide 5b S40
   7.4 Concentration-dependent libraries of peptide 5c S42
   7.5 CD spectra of isolated monomers and dimers from DCLs generated by peptides 5a-c S43

7.5.1 Monomer and dimers of peptide 5a S43
7.5.2 Monomer and dimers of peptide 5b S44
7.5.3 Monomer and dimers of peptide 5c S45

7.6 IM-MS of isolated monomers and dimers from DCLs generated by peptides 5a-c S46
7.7 MD simulations of dimers $5a_2$ and $5b_2$ S47

8. Libraries generated from tetrapeptides S48

9. 1H NMR spectra of the new peptides S49

9.1 Peptides 1a and 1b S49
9.2 Peptides 2a and 2b S50
9.3 Peptide 3b.. S51
9.4 Peptide 4b.. S51
9.5 Peptides 5b and 5c S52
9.6 Peptides 6a, 6b and 6c.. S53

10. References.. S54

1. Methods

All reagents and solvents were purchased from commercial sources (Sigma-Aldrich, Fluorochem, Tokyo Chemical Industry Co, acrGmbH) and were used without further purification.

UV-Vis analyses. Resin loading was calculated based on published protocol using Agilent Technologies Cary 8454 UV-Vis spectrophotometer.

CD analyses. Prior to CD measurement concentration of the sample for CD experiment was established employing Agilent Technologies Cary 8454 UV-Vis spectrophotometer. CD spectra were recorded on a JASCO J-715.

Peptide purification. Peptides were purified by preparative HPLC using a Shimadzu LC-8A system equipped with a Shimadzu array detector SPD-M20A using a Gemini 10 µm C18 column 110 Å, 100*21.20 mm 10 micron from Phenomenex. Products from dynamic combinatorial library of peptide 5b were isolated using a XBridge® BEH C8 column, 130 Å, 5 µm 10*150 mm from Waters.

UHPLC-MS analyses. Purified peptides and peptide dynamic combinatorial libraries were analyzed by Ultra High-Performance Liquid Chromatography coupled with mass spectrometry using an Agilent 6530 UHPLC-QTOF system with an Agilent 1290 UHPLC and a XBridge^{TM} Premier BEH C18 2.5 µm VanGuard^{TM} FIT 21*50 mm Column or using a XSelect® Peptide CSH^{TM} C18 column, 130 Å, 3.5 µm 4.6*150 mm from Waters.

IM-MS analyses. Peptides products isolated from dynamic combinatorial libraries were analyzed by IM-MS using Agilent 6560 IM-QTOF-MS.
Methods

MD simulations. Molecular dynamics simulations of CWC-tripeptide monomers and dimers in LLL, DLD and LLD chiralities were performed using Gromacs 2023.3 software [1, 2] with Amber99sb-ildn force field. [3] Monomer systems were simulated in “closed” (disulfide bond between cysteines) and “open” (no disulfide bond between cysteines) forms. Dimer systems were simulated with (i) disulfide bonds between N-termini and C-termini (parallel) and (ii) disulfide bonds between N-terminus of the first monomer and C-terminus of the second monomer and C-terminus of the first monomer bound to N-terminus of the second monomer (antiparallel). Each system was solvated with TIP3P [4] water and simulated in periodic cubic box with 2 nm distance to the box boundary. Steepest descents energy minimization was performed prior to 1 µs production simulations performed under constant temperature and pressure (NPT) conditions using 2 fs timestep, velocity-rescale thermostat [5] (reference temperature 300 K, time constant 0.5 ps) and C-rescale barostat [6] (reference pressure 1 bar, time constant 5 ps). Lengths of bonds containing hydrogen atoms were constrained using

LINCS algorithm. [7] The particle mesh Ewald method [8] was used to calculate electrostatic interactions with a cut-off length of 1 nm and a grid spacing of 0.12 nm. Van der Waals interactions were cut off at 1 nm with potential-shift-Verlet -modifier and disperion corrections were applied for energy and pressure. For each system five replica simulations of 1 µs were performed with random initial velocities.

Abbreviation.

aa: amino acid; CD: Circular dichroism; DCL: Dynamic combinatorial library; DCM: Dichloromethane; DIPEA; N,N-Diisopropylethylamine; DMF: N,N-Dimethylformamide; DTT: Dithiothreitol; EDT: Ethane-1,2-dithiol; Fmoc: 9-Fluorenylmethoxycarbonyl; HATU: 1-[Bis(dimethylamino)methylene]-1H-1,2,3-triazolo[4,5-b]pyridinium3-oxid hexafluorophosphate; HOBt: Hydroxybenzotriazole; HPLC: High-Performance Liquid Chromatography; HR-MS: High-Resolution Mass Spectrometry; IM-MS: Ion-Mobility Mass Spectrometry; MD: Molecular dynamics; NMR: Nuclear Magnetic Resonance; SPPS: Solid-Phase Peptide Synthesis; TFA: Trifluoroacetic acid; TIS: Triisopropylsilane; TMP: 2,2,6,6-Tetramethylpiperidine; UHLC-MS: Ultra-High Performance Liquid Chromatography.

2. Solid-Phase Peptide Synthesis (SPPS)

Solid phase peptide synthesis was performed according to standard procedure described in literature. [9-11]
Resin loading was carried out using a solution of amino acid (2 eq relative to resin loading) and DIPEA (5 eq relative to resin loading) in DCM. The solution was added to 2-chlorotrityl-resin (3 mmol/g) and the mixture was agitated by a stream of air for 2.5 h. The remaining reactive groups were capped with methanol for 20 min. The resin was washed with DCM and dried.
For chain elongation the dry resin beads were swollen in 1:1 DMF/DCM for 30 min. After washing (3*DMF), the Fmoc-groups were cleaved with 20 % piperidine in DMF (3*5 min). The resin was washed with DMF (3*5 min) and, for all amino acids except cysteine, the coupling was performed by adding a solution of Fmoc-protected amino acid (5 eq, 0.6 M), HATU (5 eq, 0.2 M), HOBt (5 eq, 0.2 M) and DIPEA (10 eq.) in DMF. The mixture was agitated for 15 min. After washing with DMF (2*3 min), the coupling was repeated for 15 min. When cysteine was coupled, a solution of Fmoc-Cys(Trt)-OH (4 eq, 0.6 M), HATU (4 eq, 0.2 M), HOBt (4 eq, 0.2 M) and TMP (4 eq.) was added to the resin and stirred for 15 min. The coupling step was then performed as for the other amino acids. After washing with DMF (3*5 min), the deprotection and coupling steps were continued until the desired peptide sequence was obtained.
Before cleavage of the resin the N-terminal Fmoc-protecting group was removed with 20 % piperidine in DMF (3*5 min). The resin was washed with DMF (3*5 min) and dried. Cleavage and removal of protecting groups was performed by stirring the resin beads in a solution of TFA/H_2O/EDT/TIS = 94:2.5:2.5:1 for 2 h. After filtration, the beads were washed with DCM and MeOH and the filtrate was dried under high vacuum. The obtained crude peptides were then purified by HPLC.

Dynamic combinatorial libraries were prepared by either dissolving directly, to the desired concentration in ammonium bicarbonate buffer (50 mM, pH 8) or adding a stock solution of the peptides in H_2O + 0.01 M TFA to ammonium bicarbonate buffer (pH 8).

2.1 Peptides 1

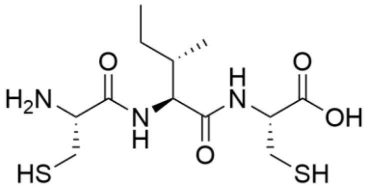

Peptide 1a was synthetized from 0.777 g of resin (loading = 0.350 mmol/g) and isolated as a white solid after preparative HPLC (gradient acetonitrile / 0.1 % TFA in H_2O = 10/90 to 95/5 in 18 min) and lyophilization (0.037 g, 40 %).

^{1}H NMR (500 MHz, D_2O, 25°C, ppm) δ^{1}H = 4.52 (t, *J*=5.5 Hz, 1H; Hα-Cys1), 4.29 (m, 2H; Hα-Ile, Hα-Cys2), 3.09 (m, 2H; Hβ-Cys2), 2.97 (m, 2H; Hβ-Cys1), 1.91 (m, 1H; Hβ-Ile), 1.53 (m, 1H; Hγ-Ile), 1.25 (m, 1H; Hγ-Ile), 0.98 (d, *J*=6.9 Hz, 3H, Hγ-Ile), 0.90 (t, *J*=7.7 Hz, 3H, Hδ-Ile). HR-MS (ESI+) calculated $C_{12}H_{24}N_3O_4S_2$ $[M+H]^+$ (*m/z*) calc. 338.1198, found 338.1201, Δ*m/z* = -0.3 mDa.

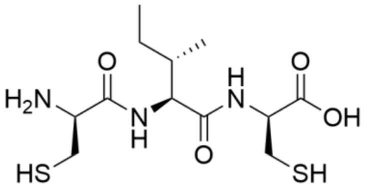

Peptide 1b was synthetized from 1.013 g of resin (loading = 0.640 mmol/g) and isolated as a white solid after by preparative HPLC (gradient acetonitrile / 0.1 % TFA in H_2O = 10/90 to 95/5 in 18 min) and lyophilization (0.050 g, 23 %).

^{1}H NMR (300 MHz, D_2O, 30°C, ppm) δ^{1}H = 4.67 (dd, *J*=6.6, 4.9 Hz, 1H; Hα-Cys1), 4.44 (d, *J*=7.5 Hz, 1H; Hα-Ile), 4.32 (t, *J*=5.9 Hz, 1H; Hα-Cys2), 3.17 (m, 2H; Hβ-Cys2), 3.07 (m, 2H; Hβ-Cys1), 2.02 (m, 1H; Hβ-Ile), 1.59 (m, 1H; Hγ-Ile), 1.32 (m, 1H; Hγ-Ile), 1.07 (d, *J*=6.8 Hz, 3H, Hγ-Ile), 0.97 (t, *J*=7.4 Hz, 3H, Hδ-Ile). HR-MS (ESI+) calculated for $C_{12}H_{24}N_3O_4S_2$ $[M+H]^+$ (*m/z*) calc. 338.1198, found 338.1224, Δ*m/z* = -2.6 mDa.

a)

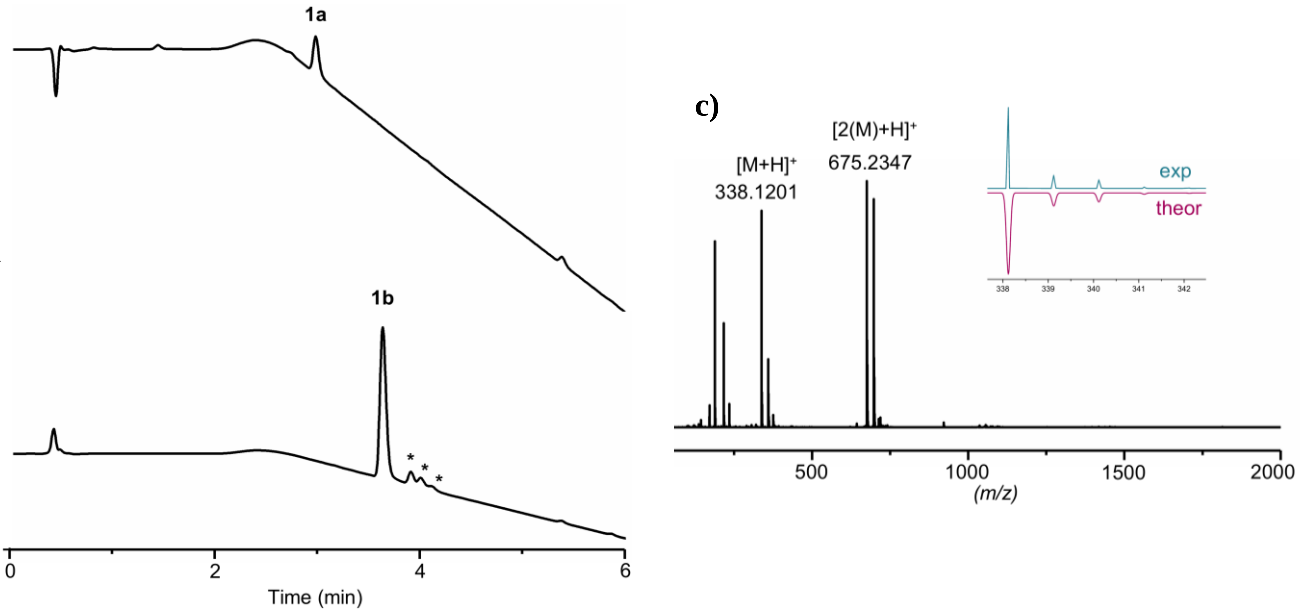

Figure S1. a) UHPLC chromatograms of purified peptides a) **1a** and b) **1b**. The ESI$^+$-MS spectrum of **1a** is shown in panel c). The ESI$^+$-MS spectrum of **1b** is identical. Elution conditions: C18 XBridge BEH, gradient acetonitrile / 0.1 % TFA in H_2O = 5/95 to 80/20 in 6 min (absorbance recorded at 220 nm). A small amount of disulfide products formed rapidly after sample preparation (peaks labeled with a star *).

2.2 Peptides 2

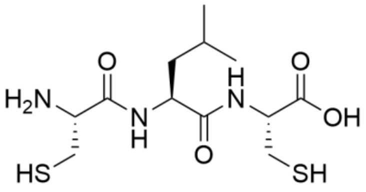

Peptide 2a was synthetized from 0.628 g of resin (loading = 0.450 mmol/g) and isolated as a white solid after preparative HPLC (gradient acetonitrile / 0.1 % TFA in H_2O = 10/90 to 95/5 in 18 min) and lyophilization (0.039 g, 41 %).

^1H NMR (300 MHz, D_2O, 30°C, ppm) δ^1H = 4.67 (dd, *J*=6.5, 4.9 Hz, 1H; Hα-Cys1), 4.55 (t, *J*=7.3 Hz, 1H; Hα-Leu), 4.33 (t, *J*=5.5 Hz, 1H; Hα-Cys2), 3.18 (dd, *J*=5.5, 4.6 Hz, 2H; Hβ-Cys2), 3.07 (dd, *J*=5.7, 4.5 Hz, 2H; Hβ-Cys1), 1.76 (m, 3H; Hβ-Leu, HγLeu), 1.02 (td, *J*=11.7, 6.0 Hz, 6H, Hδ-Leu).HR-MS (ESI+) calculated for $C_{12}H_{24}N_3O_4S_2$ $[M+H]^+$ (*m/z*) calc. 338.1198, found 338.1207, Δ*m/z* = -0.9 mDa.

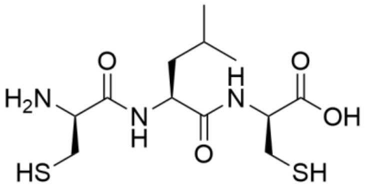

Peptide 2b was synthetized from 1.007 g of resin (loading = 0.640 mmol/g) and isolated as a white solid after preparative HPLC (gradient acetonitrile / 0.1 % TFA in H_2O = 10/90 to 95/5 in 18 min) and lyophilization (0.118 mg, 54 %).

^1H NMR (300 MHz, D_2O, 30°C, ppm) δ^1H = 4.64-4.59 (m, *J*=6.5, 4.9 Hz, 2H; Hα-Cys1, Hα-Leu), 4.32 (t, *J*=5.9 Hz, 1H; Hα-Cys2), 3.17 (dd, *J*=5.9, 3.6 Hz, 2H; Hβ-Cys2), 3.07 (m, 2H; Hβ-Cys1), 1.77 (m, 3H; Hβ-Leu, HγLeu), 1.02 (td, *J*=11.9, 5.8 Hz, 6H, Hδ-Leu). HR-MS (ESI+) calculated for $C_{12}H_{24}N_3O_4S_2$ $[M+H]^+$ (*m/z*) calc. 338.1198, found 338.1214, Δ*m/z* = -1.6 mDa.

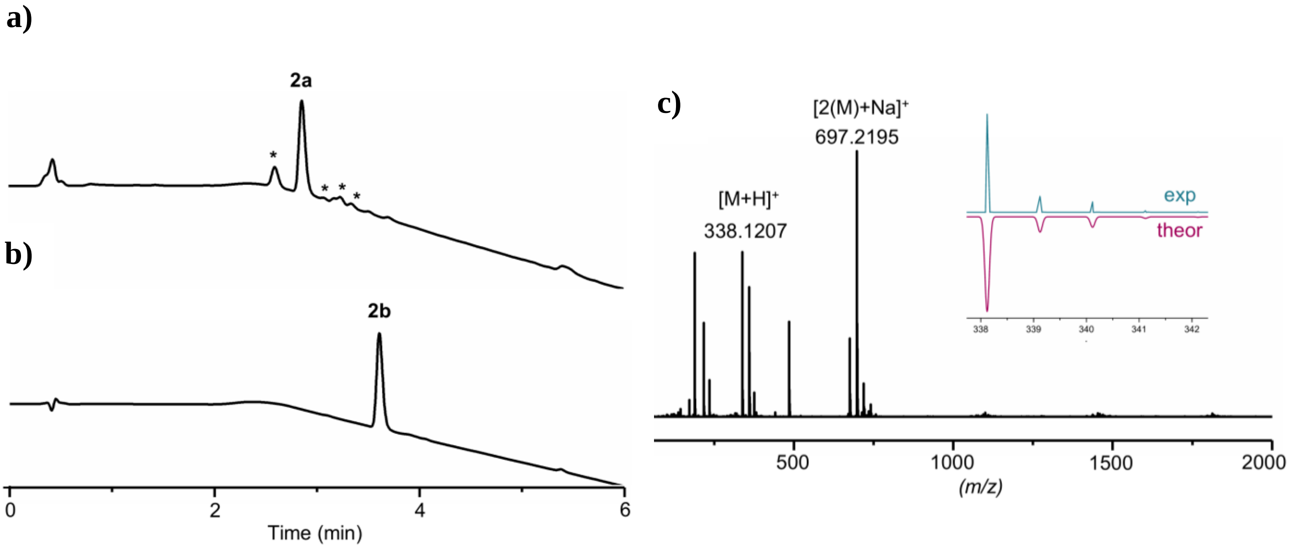

Figure S2. UHPLC chromatograms of purified peptides a) **2a** and b) **2b**. The ESI^+-MS spectrum of **2a** is shown in panel c). The ESI^+-MS spectrum of **2b** is identical. Elution conditions: C18 XBridge BEH, gradient acetonitrile / 0.1 % TFA in H_2O = 5/95 to 80/20 in 6 min (absorbance recorded at 220 nm). A small amount of disulfide products formed rapidly after sample preparation (peaks labeled with a star *).

2.3 Peptides 3

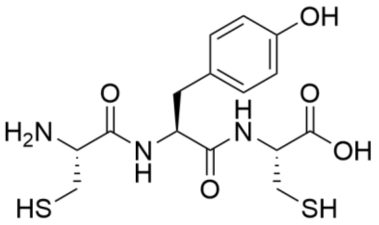

Peptide 3a was synthetized from 1.080 g of resin (loading = 0.200 mmol/g) and isolated as a white solid after preparative HPLC (gradient acetonitrile / 0.1 % TFA in H_2O = 10/90 to 95/5 in 18 min) and lyophilization (0.026 mg, 31 %).
^{1}H NMR spectrum closely matches that previously reported in literature [12]. HR-MS (ESI+) calculated for $C_{15}H_{22}N_3O_5S_2$ $[M+H]^+$ (*m/z*) calc. 388.0991, found 388.0990, Δ*m/z* = 0.1 mDa.

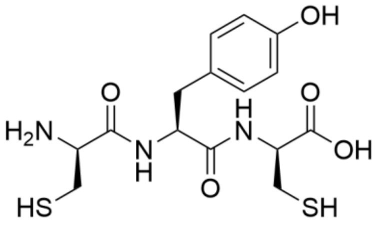

Peptide 3b was synthetized from 0.998 g of resin (loading = 0.640 mmol/g) and isolated as a white solid after preparative HPLC (gradient acetonitrile / 0.1 % TFA in H_2O = 10/90 to 95/5 in 18 min) and lyophilization (0.106 mg, 43 %).
^{1}H NMR (300 MHz, D_2O, 30°C, ppm) δ^1H = 7.29 (d, *J*=8.6 Hz, 2H; Haro-Tyr), 6.69 (d, *J*=8.6 Hz, 2H; Haro-Tyr), 4.86 (m, 1H; Hα-Phe), 4.53 (dd, *J*=6.1, 4.4 Hz, 1H; Hα-Cys1), 4.28 (t, *J*=5.3 Hz, 1H; Hα-Cys2), 3.26 (m, 1H; Hβ-Tyr), 3.09 (m, 1H; Hβ-Tyr), 2.94 (m, 4H; Hβ-Cys). HR-MS (ESI+) calculated for $C_{15}H_{22}N_3O_5S_2$ $[M+H]^+$ (*m/z*) calc. 388.0991, found 388.0999, Δ*m/z* = -0.8 mDa.

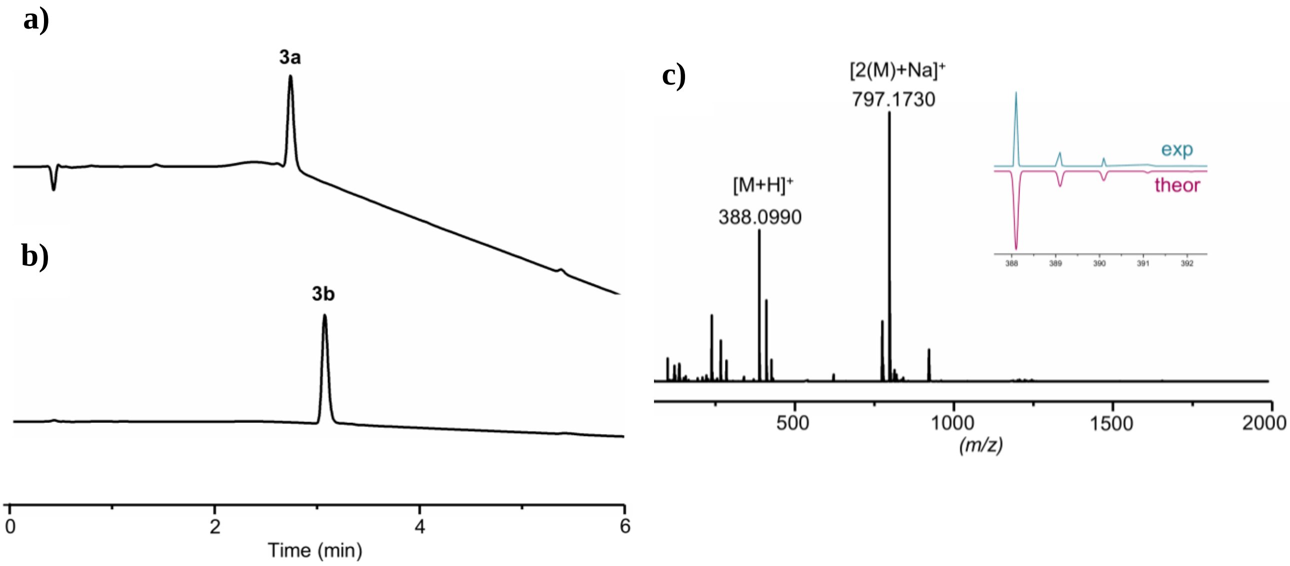

Figure S3. UHPLC chromatograms of purified peptides a) **3a** and b) **3b**. The ESI$^+$-MS spectrum of **3a** is shown in panel c). The ESI$^+$-MS spectrum of **3b** is identical. Elution conditions: C18 XBridge BEH, gradient acetonitrile / 0.1 % TFA in H_2O = 5/95 to 80/20 in 6 min (absorbance recorded at 220 nm).

2.4 Peptides 4

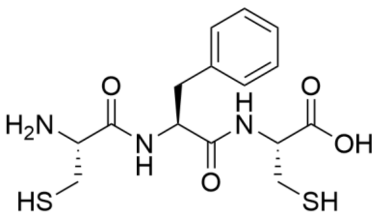

Peptide 4a was synthetized from 0.930 g of resin (loading = 0.450 mmol/g) and isolated as a white solid after preparative HPLC (gradient acetonitrile / 0.1 % TFA in H_2O = 10/90 to 95/5 in 18 min) and lyophilization (0.049 mg, 31 %).
^1H NMR spectrum closely matches that previously reported in literature [12]. HR-MS (ESI+) calculated for $C_{15}H_{22}N_3O_4S_2$ $[M+H]^+$ (*m/z*) calc. 372.1051, found 372.1045, $\Delta m/z$ = 0.6 mDa .

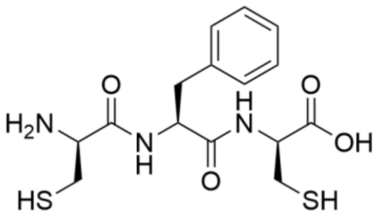

Peptide 4b was synthetized from 1.001 g of resin (loading = 0.640 mmol/g) and isolated as a white solid after preparative HPLC (gradient acetonitrile / 0.1 % TFA in H_2O = 10/90 to 95/5 in 18 min) and lyophilization to yield (0.061 g, 26 %).
^1H NMR (300 MHz, D_2O, 30°C, ppm) δ^1H = 7.48 (m, 2H; Haro-Phe), 7.43 (m, 3H; Haro-Phe), 4.93 (dd, *J*=9.5, 6.4 Hz, 1H; Hα-Phe), 4.61 (dd, *J*=6.2, 4.7 Hz, 1H; Hα-Cys1), 4.26 (t, *J*=5.5 Hz, 1H; Hα-Cys2), 3.32 (m, 1H; Hβ-Phe), 3.10 (m, 1H; Hβ-Phe), 2.93 (m, 4H; Hβ-Cys). HR-MS (ESI+) calculated for $C_{15}H_{22}N_3O_4S_2$ $[M+H]^+$ (*m/z*) calc. 372.1051, found 372.1051, $\Delta m/z$ = 0 mDa.

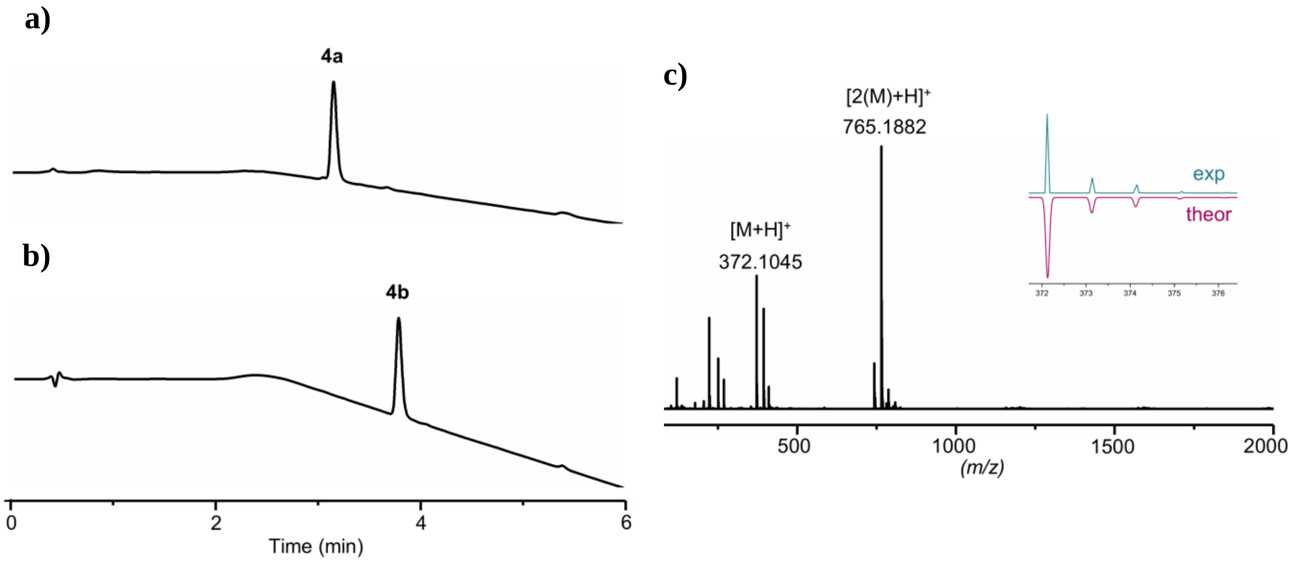

Figure S4. a) UHPLC chromatograms of purified peptides a) **4a** and b) **4b**. The ESI$^+$-MS spectrum of **4a** is shown in panel c). The ESI$^+$-MS spectrum of **4b** is identical. Elution conditions: C18 XBridge BEH, gradient acetonitrile / 0.1 % TFA in H_2O = 5/95 to 80/20 in 6 min (absorbance recorded at 220 nm).

2.5 Peptides 5

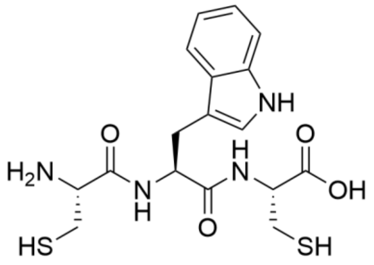

Peptide 5a was synthetized from 1.067 g of resin (loading = 0.200 mmol/g) and isolated as a white solid after preparative HPLC (gradient acetonitrile / 0.1 % TFA in H_2O = 5/95 to 95/5 in 18 min) and lyophilization to yield (0.025 g, 29 %).
^{1}H NMR spectrum closely matches that previously reported in literature [12]. HR-MS (ESI+) calculated for $C_{17}H_{23}N_4O_4S_2$ $[M+H]^+$ (*m/z*) calc. 411.1150, found 411.1151, Δ*m/z* = -0.1 mDa.

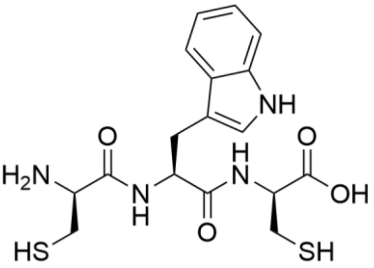

Peptide 5b was synthetized from 0.966 g of resin (loading = 0.640 mmol/g) and isolated as a white solid after preparative HPLC (gradient acetonitrile / 0.1 % TFA in H_2O = 10/90 to 95/5 in 18 min) and lyophilization (0.044 g, 17 %).
^{1}H NMR (300 MHz, D_2O, 30°C, ppm) δ^{1}H = 7.79 (d, *J*=7.7, 1H; Hζ′-Trp), 7.59 (d, *J*=8.1, 1H; Hε-Trp), 7.38 (s, 1H; Hδ-Trp), 7.31 (m, 1H; Hζ-Trp), 7.25 (m, 1H; Hη-Trp), 4.98 (t, *J*=8.0 Hz, 1H; Hα-Trp), 4.55 (dd, *J*=6.3, 4.5 Hz, 1H; Hα-Cys1), 4.25 (t, *J*=5.5 Hz, 1H; Hα-Cys2), 3.45 (m, 1H; Hβ-Trp), 3.33 (m, 1H; Hβ-Trp), 2.88-2.81 (m, 3H; Hβ-Cys2, Hβ-Cys1), 2.67 (m, 1H; Hβ-Cys1). HR-MS (ESI+) calculated for $C_{17}H_{23}N_4O_4S_2$ $[M+H]^+$ (*m/z*) calc. 411.1150, found 411.1156, Δ*m/z* = -0.6 mDa.

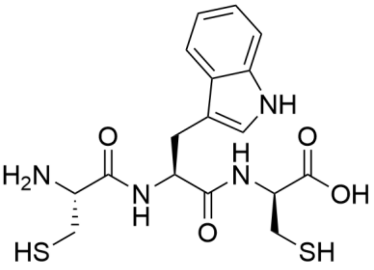

Peptide 5c was synthetized from 1.022 g of resin (loading = 0.640 mmol/g) and isolated as a white solid after preparative HPLC (gradient acetonitrile / 0.1 % TFA in H_2O = 10/90 to 85/5 in 18 min) and lyophilization (0.038 g, 48 %).
^{1}H NMR (500 MHz, D_2O, 25°C, ppm) δ^{1}H = 7.68 (d, *J*=7.5, 1H; Hζ′-Trp), 7.51 (d, *J*=8.1, 1H; Hε-Trp), 7.30 (s, 1H; Hδ-Trp), 7.26 (t, *J*=7.9 Hz, 1H; Hζ-Trp), 7.18 (t, *J*=7.8 Hz, 1H; Hη-Trp), 4.74 (m, 1H; Hα-Trp), 4.41 (m, 1H; Hα-Cys1), 4.25 (m, 1H; Hα-Cys2), 3.31 (m, 2H; Hβ-Trp), 3.10 (m, 2H; Hβ-Cys2), 2.58 (m, 1H; Hβ-Cys1), 2.23 (m, 1H; Hβ-Cys1). HR-MS (ESI+) calculated for $C_{17}H_{23}N_4O_4S_2$ $[M+H]^+$ (*m/z*) calc. 411.1150, found 411.1174, Δ*m/z* = 2.4 mDa.

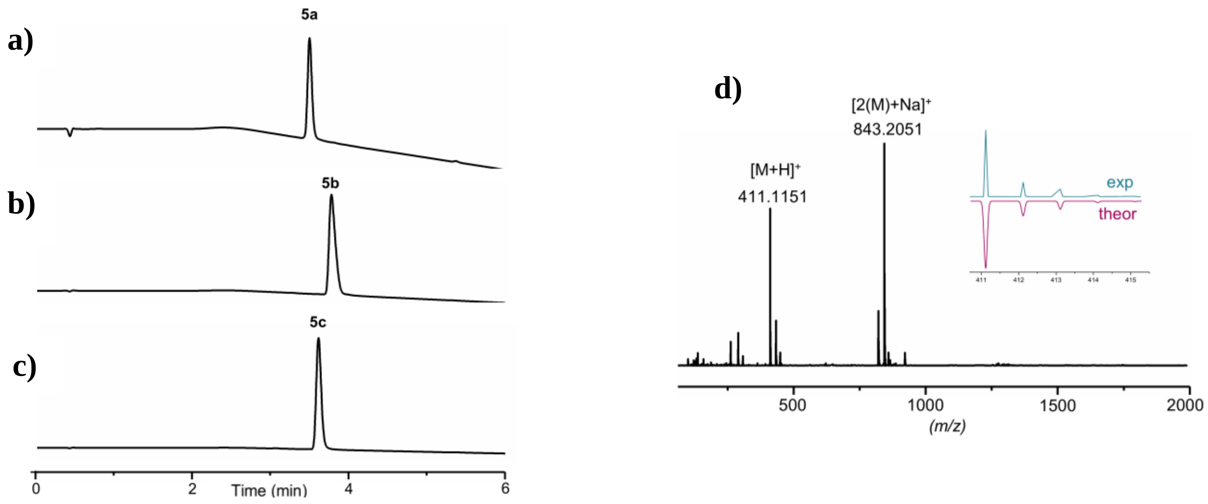

Figure S5. a) UHPLC chromatograms of purified peptides a) **5a**, b) **5b** and c) **5c**. The ESI$^+$-MS spectrum of **5a** is shown in panel d), the ESI$^+$-MS spectra of **5b** and **5c** are identical. Elution conditions: C18 XBridge BEH, gradient acetonitrile / 0.1 % TFA in H_2O = 5/95 to 80/20 in 6 min (absorbance recorded at 220 nm).

2.6 Peptides 6

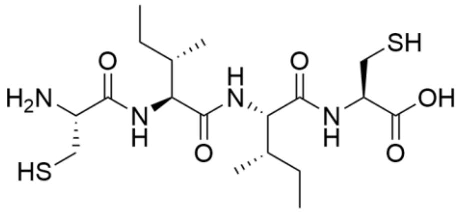

Peptide 6a was synthetized from 1.009 g of resin (loading = 0.450 mmol/g) and isolated as a white solid after preparative HPLC (gradient Acetonitrile / 0.1 % TFA in H_2O = 10/90 to 95/5 in 18 min) and lyophilization (0.125 g, 61 %).

^{1}H NMR (500 MHz, D_2O, 25°C, ppm) δ^1H = 4.58 (t, *J*=5.7 Hz, 1H; Hα-Cys1), 4.28-4.26 (m, 3H; Hα-Ile, Hα-Cys2), 3.08 (t, *J*=5.2 Hz, 2H; Hβ-Cys2), 2.97 (m, 2H; Hβ-Cys1), 1.88 (m, 2H; Hβ-Ile), 1.52 (m, 2H; Hγ-Ile), 1.21 (m, 2H; Hγ-Ile), 0.95 (d, *J*=6.7 Hz, 3H, Hγ-Ile), 0.92 (d, *J*=6.7 Hz, 3H, Hγ-Ile), 0.90 (m, 6H, Hδ-Ile). HR-MS (ESI+) calculated for $C_{18}H_{35}N_4O_5S_2$ $[M+H]^+$ (*m/z*) calc. 451.2040, found 451.2065, Δ*m/z* = -2.5 mDa.

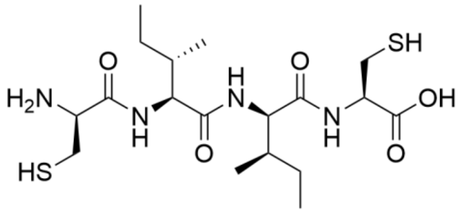

Peptide 6b was synthetized from 1.000 g of resin (loading = 0.640 mmol/g) and isolated as a white solid after preparative HPLC (gradient acetonitrile / 0.1 % TFA in H_2O = 10/90 to 95/5 in 18 min) and lyophilization (0.085 g, 30 %).

^{1}H NMR (500 MHz, D_2O, 25°C, ppm) δ^1H = 4.57 (m, 1H; Hα-Cys1), 4.31 (d, *J*=8.0 Hz, 1H; Hα-Ile), 4.22 (m, 2H; Hα-Ile, Hα-Cys2), 3.07 (m, 4H; Hβ-Cys2, Hβ-Cys1), 1.95 (m, 1H; Hβ-Ile), 1.89 (m, 1H; Hβ-Ile), 1.51 (m, 2H; Hγ-Ile), 1.24 (m, 2H; Hγ-Ile), 0.96 (m, 6H, Hγ-Ile), 0.89 (m, 6H, Hδ-Ile). HR-MS (ESI+) calculated for $C_{18}H_{35}N_4O_5S_2$ $[M+H]^+$ (*m/z*) calc. 451.2040, found 451.2064, Δ*m/z* = -2.4 mDa.

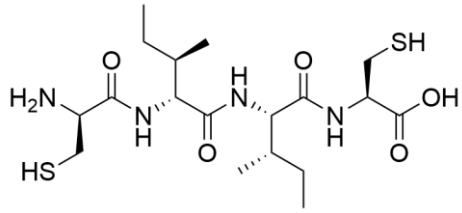

Peptide 6c was synthetized from 1.000 g of resin (loading = 0.640 mmol/g) and isolated as a white solid after preparative HPLC (gradient acetonitrile / 0.1 % TFA in H_2O = 10/90 to 95/5 in 18 min) and lyophilization (0.125 g, 43 %).

^{1}H NMR (500 MHz, D_2O, 25°C, ppm) δ^1H = 4.56 (t, *J*=5.8 Hz, 1H; Hα-Cys1), 4.30 (d, *J*=8.1 Hz, 1H; Hα-Ile), 4.26 (t, *J*=5.6 Hz, 1H; Hα-Cys2), 4.23 (d, *J*=8.4 Hz, 1H, Hα-Ile), 3.06 (dd, *J*=11.1, 5.7 Hz, 2H; Hβ-Cys2), 2.98 (m, 2H; Hβ-Cys1), 1.94 (m, 1H; Hβ-Ile), 1.88 (m, 1H; Hβ-Ile), 1.50 (m, 2H; Hγ-Ile), 1.23 (m, 2H; Hγ-Ile), 0.96 (m, 6H, Hγ-Ile), 0.88 (m, 6H, Hδ-Ile). HR-MS (ESI+) calculated for $C_{18}H_{35}N_4O_5S_2$ $[M+H]^+$ (*m/z*) calc. 451.2040, found 451.2067, Δ*m/z* = 2.7 mDa.

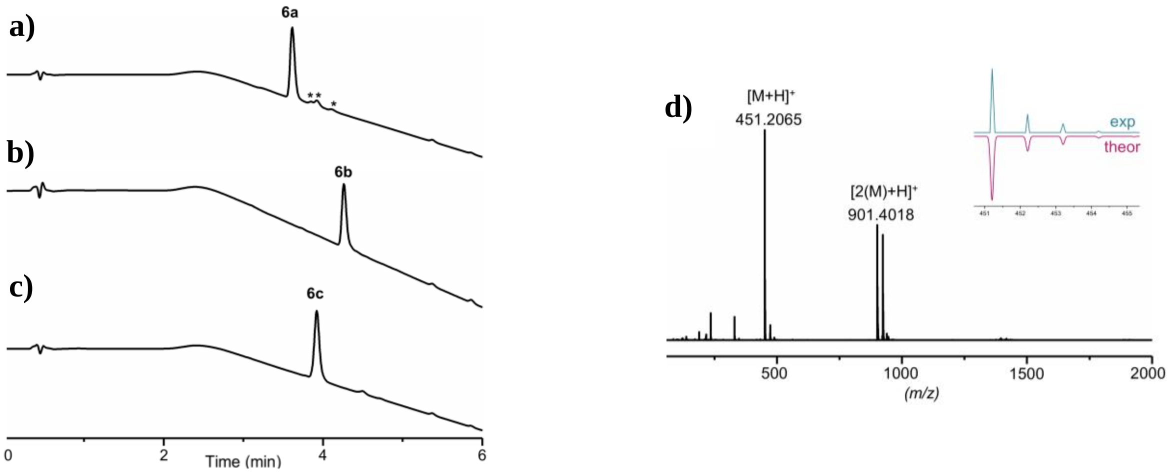

Figure S6. UHPLC chromatograms of purified peptides a) **6a**, b) **6b** and c) **6c**. The ESI$^+$-MS spectrum of **6a** is shown in panel d). The ESI$^+$-MS spectra of **6b** and **6c** are identical.. Elution conditions: C18 XBridge BEH, gradient acetonitrile / 0.1 % TFA in H_2O = 5/95 to 80/20 in 6 min (absorbance recorded at 220 nm). A small amount of disulfide products formed rapidly after sample preparation (peaks labeled with a star *).

3. Reversibility test and reaction time optimization

To assess the reversibility of the dynamic combinatorial process, 5 % of DTT was added to a preequilibrated library generated from peptide **1b**. The partially reduced library contained a mixture of fully reduced peptide **1b**, partially oxidized products, and fully oxidized closed monomer. Small amounts of closed dimers products were also observed. The library was then left to re-oxidize in air for 24 more hours. The composition of the re-equilibrated library was identical to that originally obtained directly from **1b**, as expected from a system under thermodynamic control.

a)

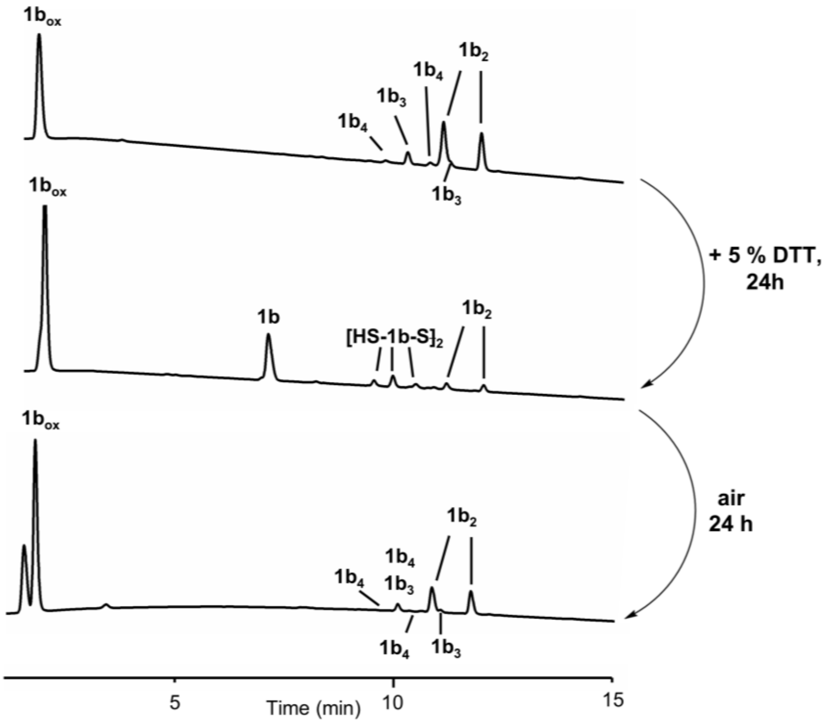

b) Intermediate oxidized product [HS-1b-S]₂

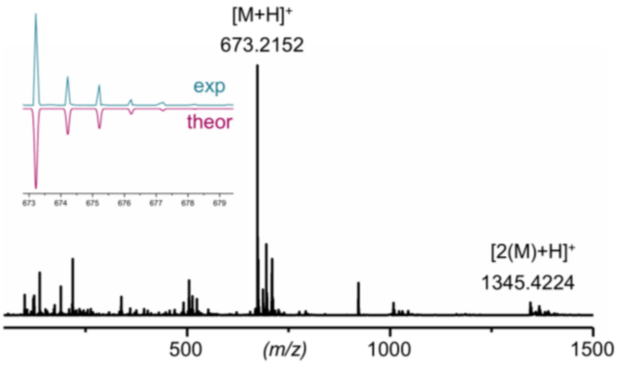

Figure S7. a) UHPLC chromatograms of the libraries generated from peptide **1b** (10 mM) to test disulfide reversibility and b) ESI^+-MS spectrum of the intermediate oxidized product observed 24 h after addition of 5 % DTT. Elution conditions: C18 XBridge BEH, gradient acetonitrile / 0.1 % TFA in H_2O = 5/95 to 80/20 in 30 min (absorbance recorded at 220 nm). The peak labelled **1b**$_{ox}$ in the chromatograms correspond to the sodium adduct [**1b**$_{ox}$+Na]$^+$ and the species eluting earlier corresponds to the protonated closed monomer [**1b**$_{ox}$ + H]$^+$.

Thermodynamic equilibrium was reached within approximately 48 h for all the libraries. After 3 days, the formation of undesired overoxidized sulfoxide and sulfone type products was observed.

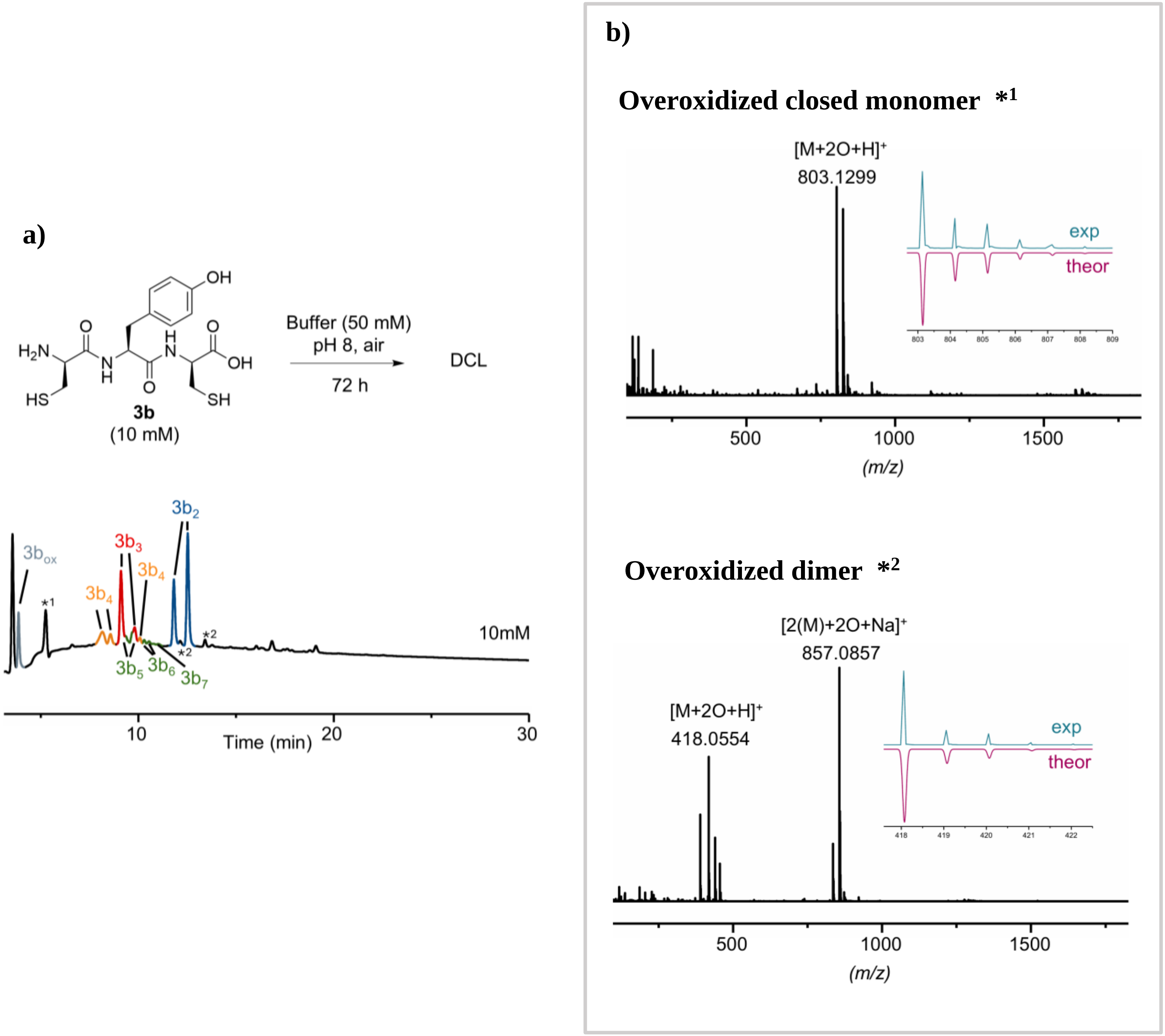

Figure S8. a) UHPLC chromatogram of the library generated from peptide **3b** (10 mM) after 3 days. C18 XSelect peptide CSH column, gradient acetonitrile / 0.1 % TFA in H_2O = 15/85 to 40/60 in 30 min (absorbance recorded at 220 nm). Overoxidized monomer and dimers are labelled with (*[1]) and (*[2]) respectively. b) ESI$^+$-MS spectra of the corresponding overoxidized products found in the library.

4. Libraries generated from homochiral LLL-tripeptides

4.1 Concentration-dependent libraries

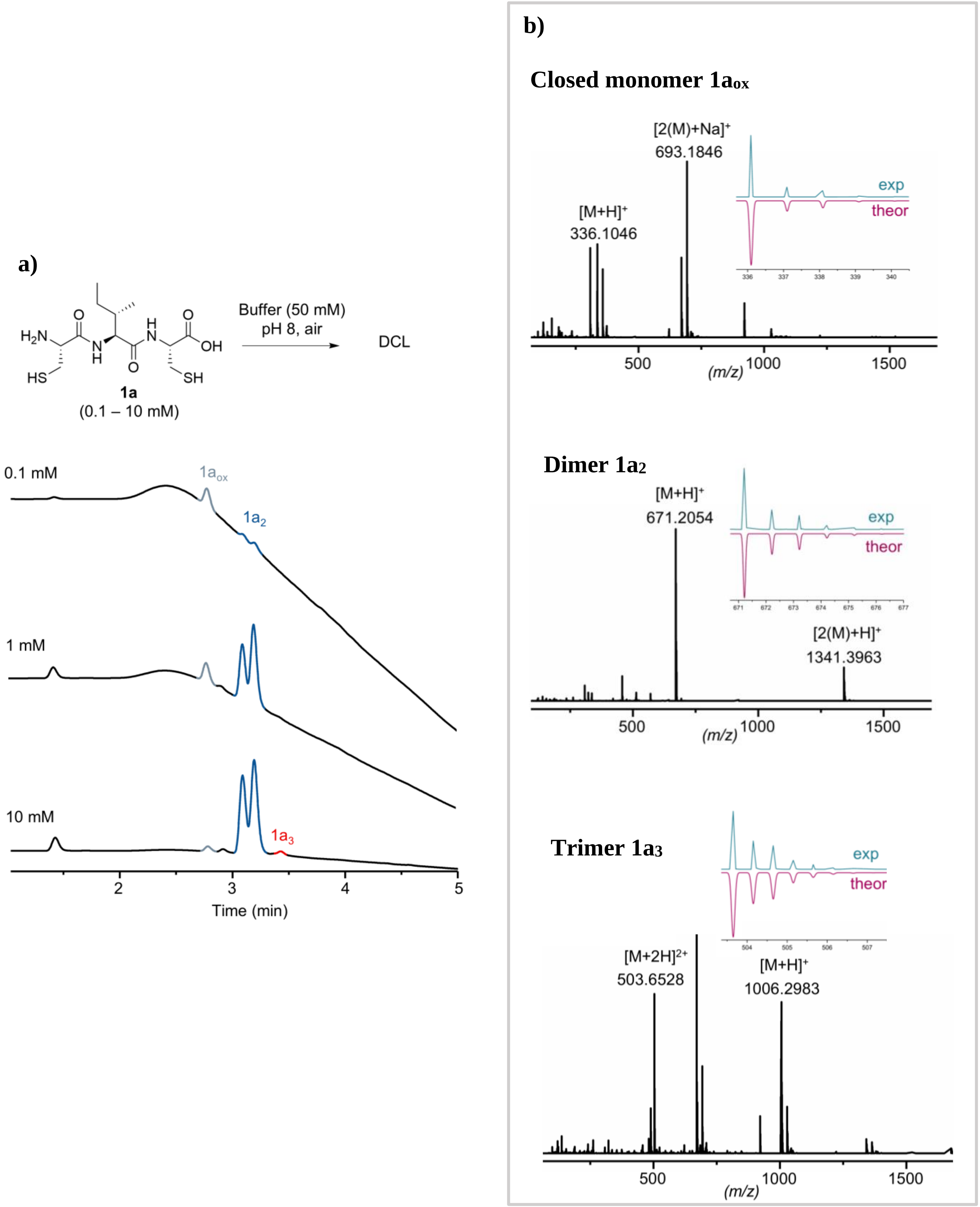

Figure S9. a) UHPLC chromatograms of the libraries generated from peptide **1a** at different concentration. Elution conditions: C18 XBridge BEH, gradient acetonitrile / 0.1 % TFA in H_2O = 5/95 to 80/20 in 6 min (absorbance recorded at 220 nm). b) ESI^+-MS spectra of the corresponding oxidized products found in the libraries.

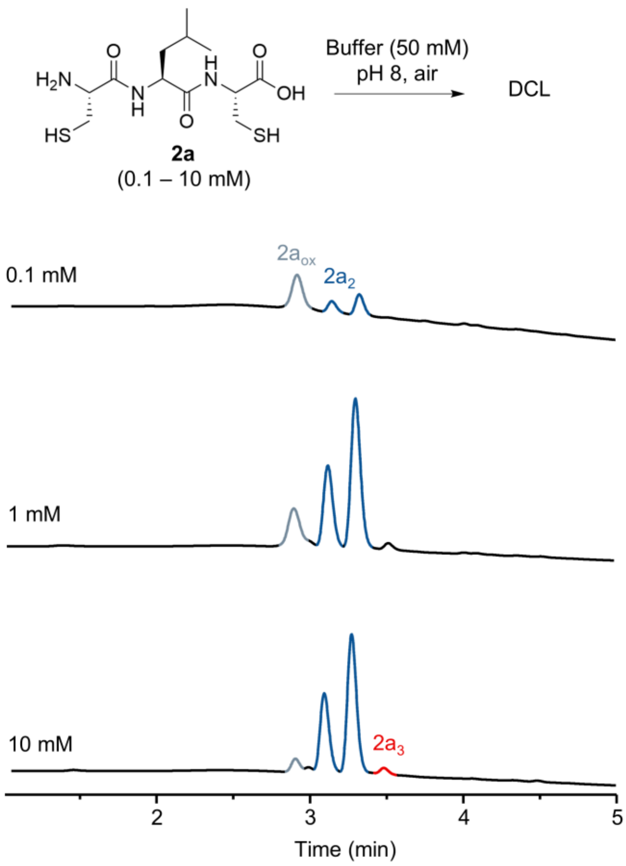

Figure S10. a) UHPLC chromatograms of the libraries generated from peptide **2a** at different concentration. Elution conditions: C18 XBridge BEH, gradient acetonitrile / 0.1 % TFA in H_2O = 5/95 to 80/20 in 6 min (absorbance recorded at 220 nm). Since peptides **1a** and **2a** have the same mass, the ESI^+-MS spectrum of the corresponding oxidized products are identical to the one already displayed in figure **S9**.

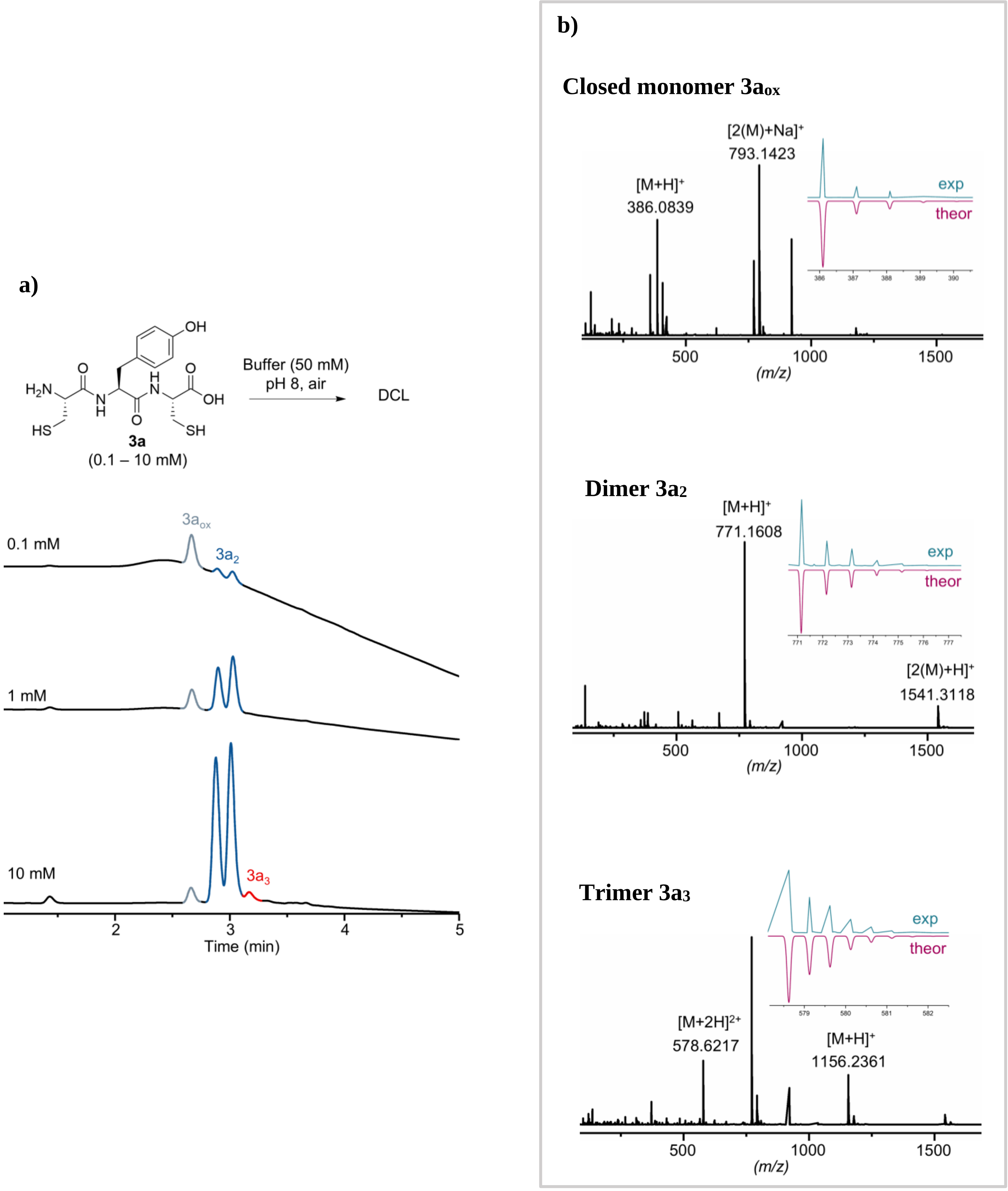

Figure S11. a) UHPLC chromatograms of the libraries generated from peptide **3a** at different concentration. Elution conditions: C18 XBridge BEH, gradient acetonitrile / 0.1 % TFA in H_2O = 5/95 to 80/20 in 6 min (absorbance recorded at 220 nm). b) ESI^+-MS spectra of the corresponding oxidized products found in the libraries.

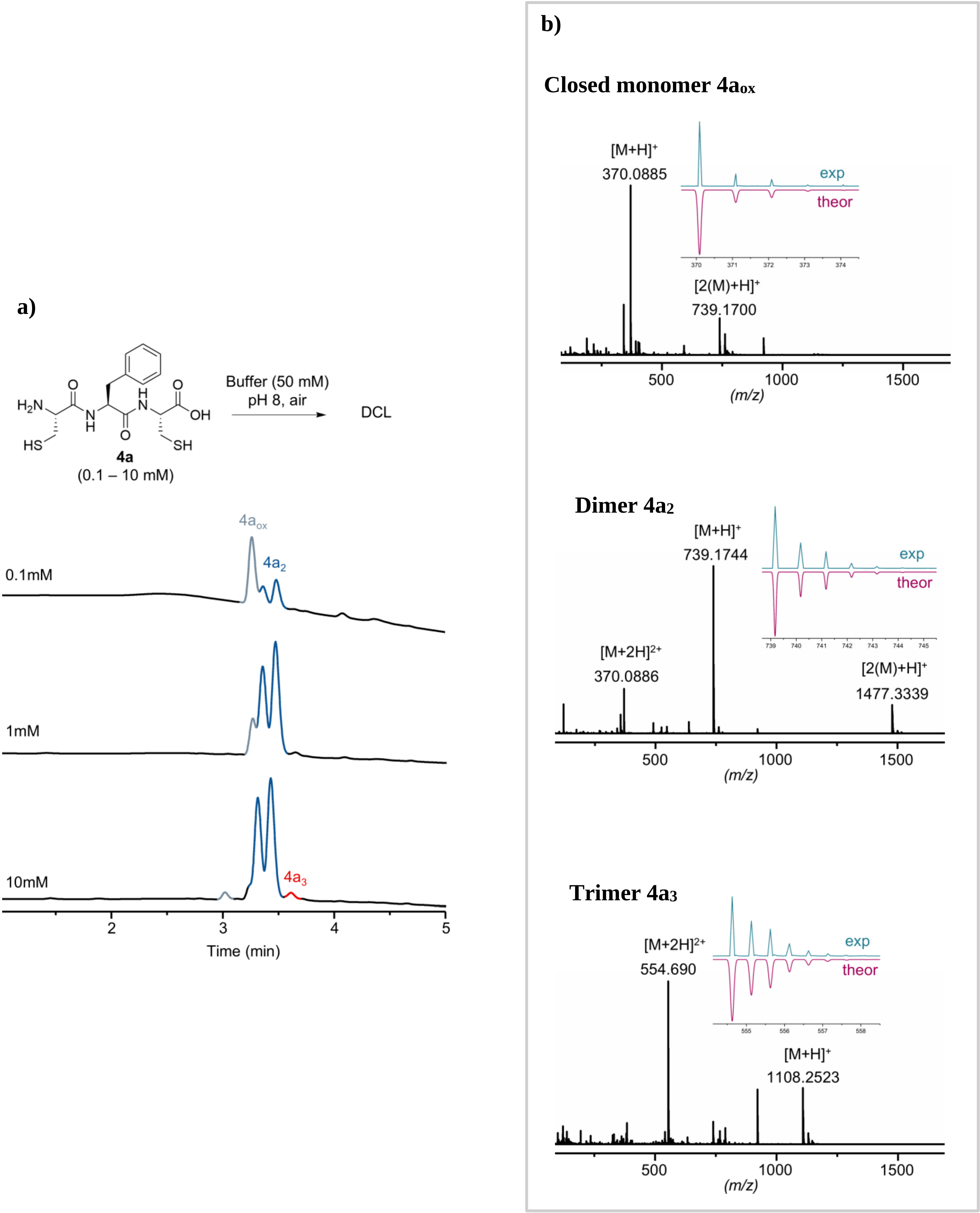

Figure S12. a) UHPLC chromatograms of the libraries generated from peptide **4a** at different concentration. Elution conditions: C18 XBridge BEH, gradient acetonitrile / 0.1 % TFA in H_2O = 5/95 to 80/20 in 6 min (absorbance recorded at 220 nm). b) ESI^+-MS spectra of the corresponding oxidized products found in the libraries.

4.2 Effect of high salt concentration (1M NaCl)

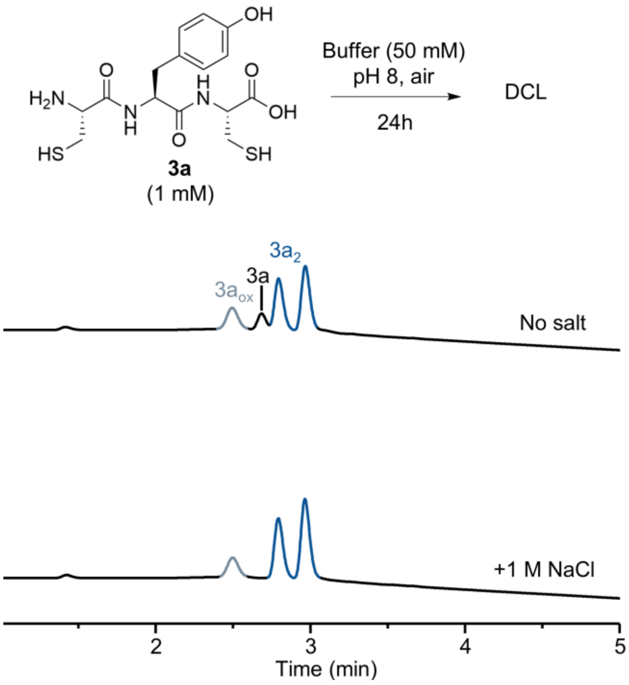

Figure S13. UHPLC chromatograms of the libraries generated from peptide **3a** (1 mM) without and with 1M of NaCl after 24h. Elution conditions: C18 XBridge BEH, gradient acetonitrile / 0.1 % TFA in H_2O = 5/95 to 80/20 in 6 min (absorbance recorded at 220 nm). In this case, the addition of salt accelerates the formation of disulfide bonds but does not change the library composition.

5. Libraries generated from heterochiral DLD-tripeptides

5.1 Comparison of libraries generated from homochiral LLL- and heterochiral DLD-tripeptides and concentration-dependent libraries

5.1.1 Peptide 1b

a)

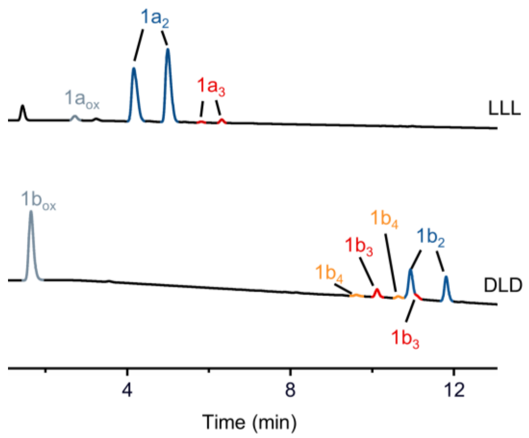

b) Tetramer 1b4

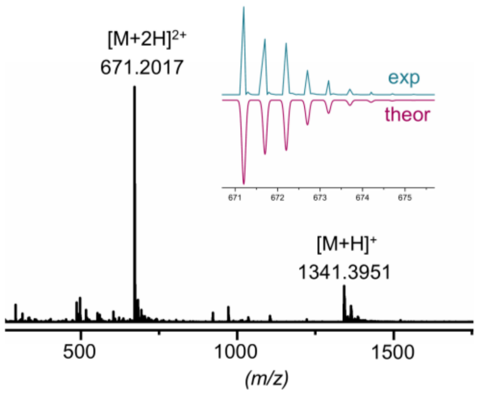

Figure S14. a) UHPLC chromatograms comparison of the libraries generated from the homochiral peptide **1a** and the heterochiral peptide **1b** (10 mM). Elution conditions: C18 XBridge BEH, gradient acetonitrile / 0.1 % TFA in H_2O = 5/95 to 80/20 in 30 min (absorbance recorded at 220 nm). b) ESI$^+$-MS spectrum of the corresponding new oxidized product found in the library.

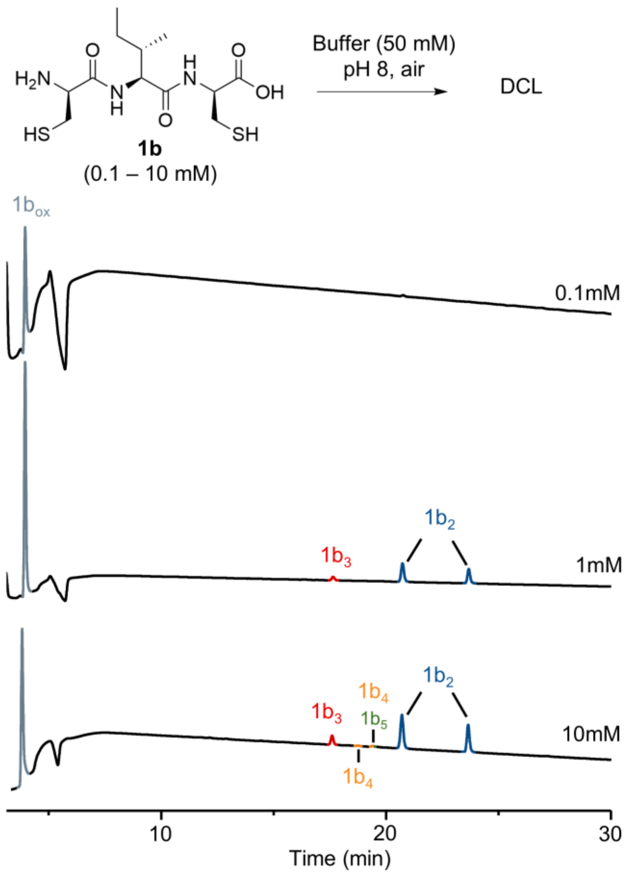

Figure S15. UHPLC chromatograms of the libraries generated from peptide **1b** at different concentration. Elution conditions: C18 XSelect peptide CSH, gradient acetonitrile / 0.1 % TFA in H_2O = 15/85 to 40/60 in 30 min (absorbance recorded at 220 nm).

5.1.2 Peptide 2b

a) **b) Pentamer 2b5**

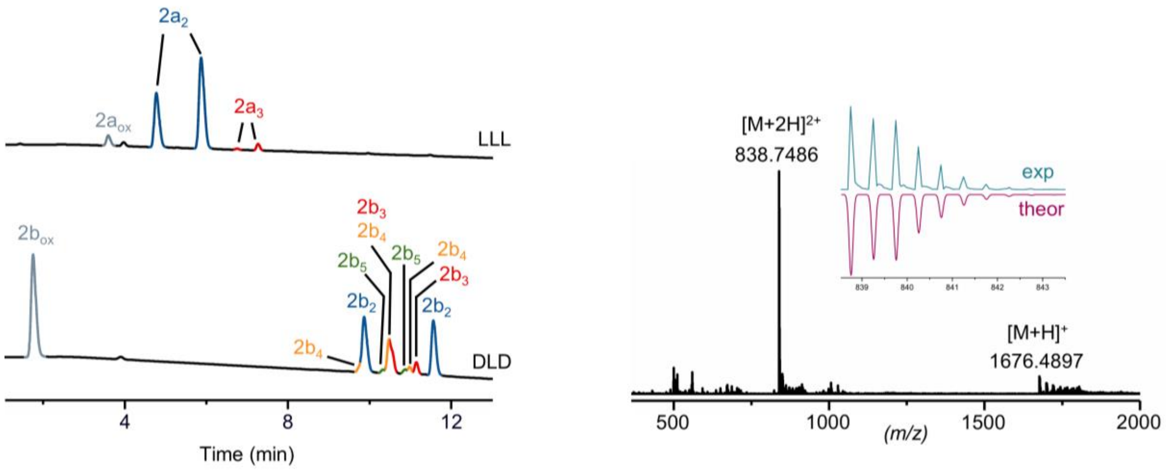

Figure S16. a) UHPLC chromatograms comparison of the libraries generated from the homochiral peptide **2a** and the heterochiral peptide **2b** (10 mM). Elution conditions: C18 XBridge BEH, gradient acetonitrile / 0.1 % TFA in H_2O = 5/95 to 80/20 in 30 min (absorbance recorded at 220 nm). b) ESI^+-MS spectrum of the corresponding new oxidized product found in the library.

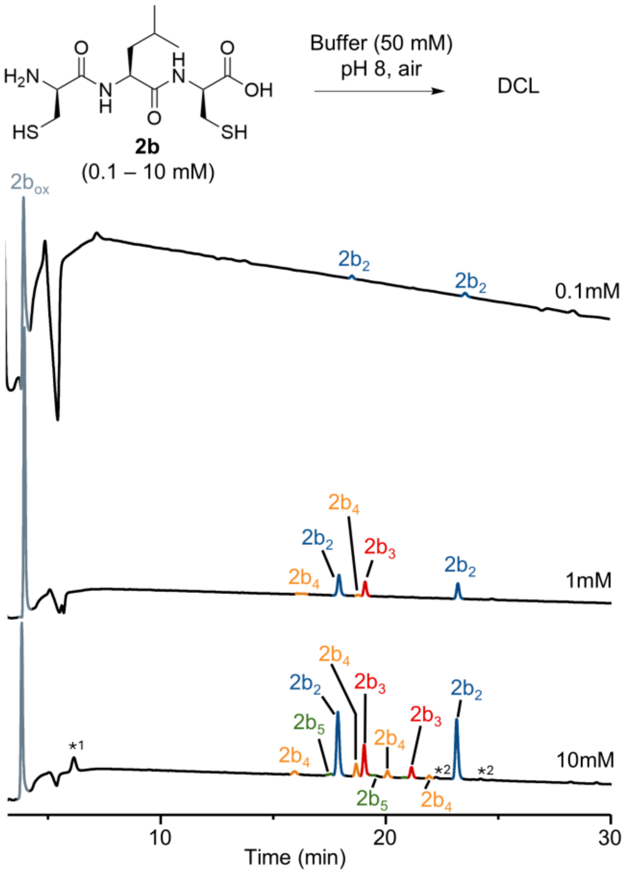

Figure S17. UHPLC chromatograms of the libraries generated from peptide **2b** at different concentration. Elution conditions: C18 XSelect peptide CSH, gradient acetonitrile / 0.1 % TFA in H_2O = 15/85 to 40/60 in 30 min (absorbance recorded at 220 nm). Overoxidized monomer and dimers are labelled with (*[1]) and (*[2]) respectively.

5.1.3 Peptide 3b

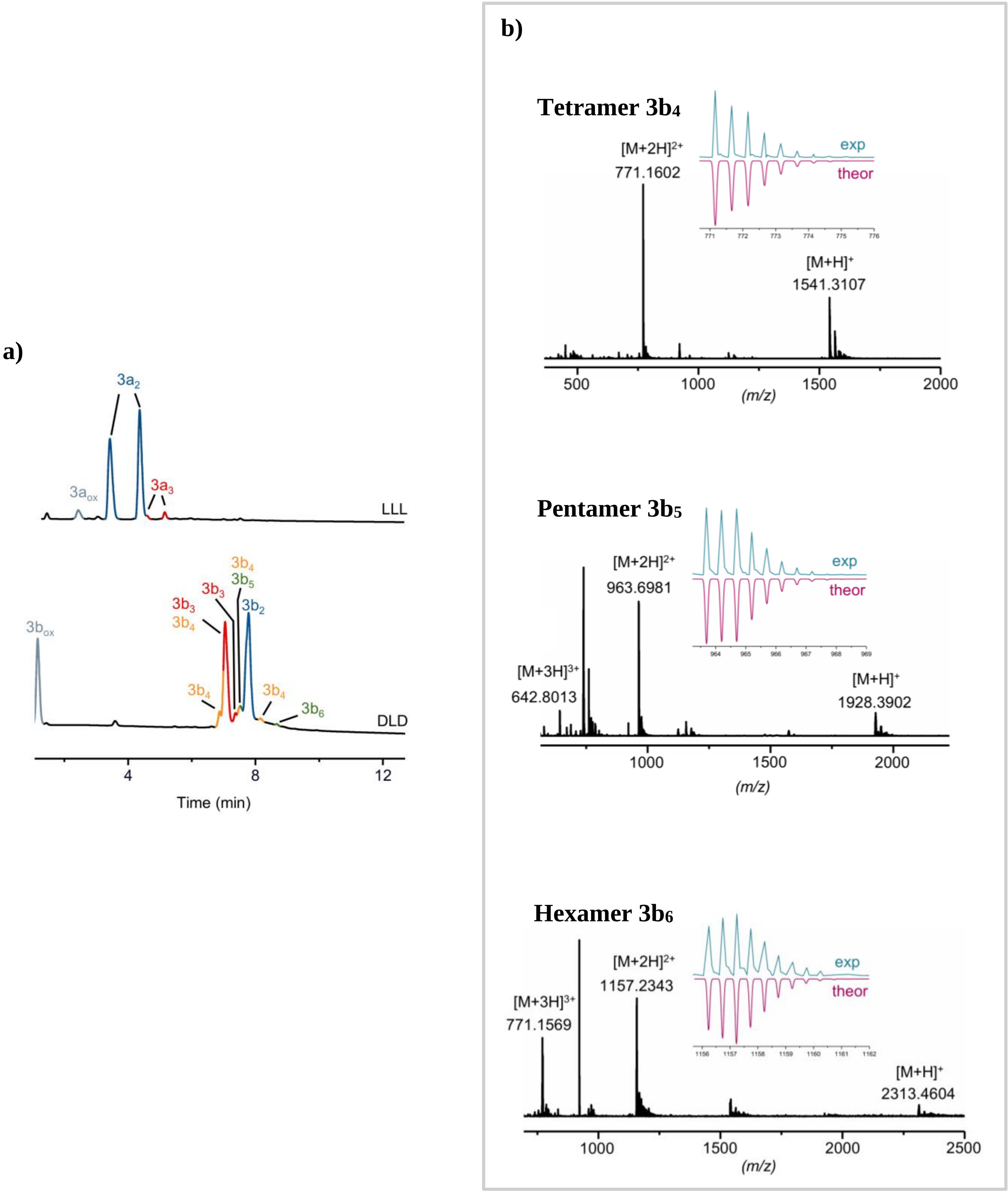

Figure S18. a) UHPLC chromatograms comparison of the libraries generated from the homochiral peptide **3a** and the heterochiral peptide **3b** (10 mM). Elution conditions: C18 XBridge BEH, gradient acetonitrile / 0.1 % TFA in H_2O = 5/95 to 80/20 in 30 min (absorbance recorded at 220 nm). b) ESI^+-MS spectra of the corresponding new oxidized products found in the library.

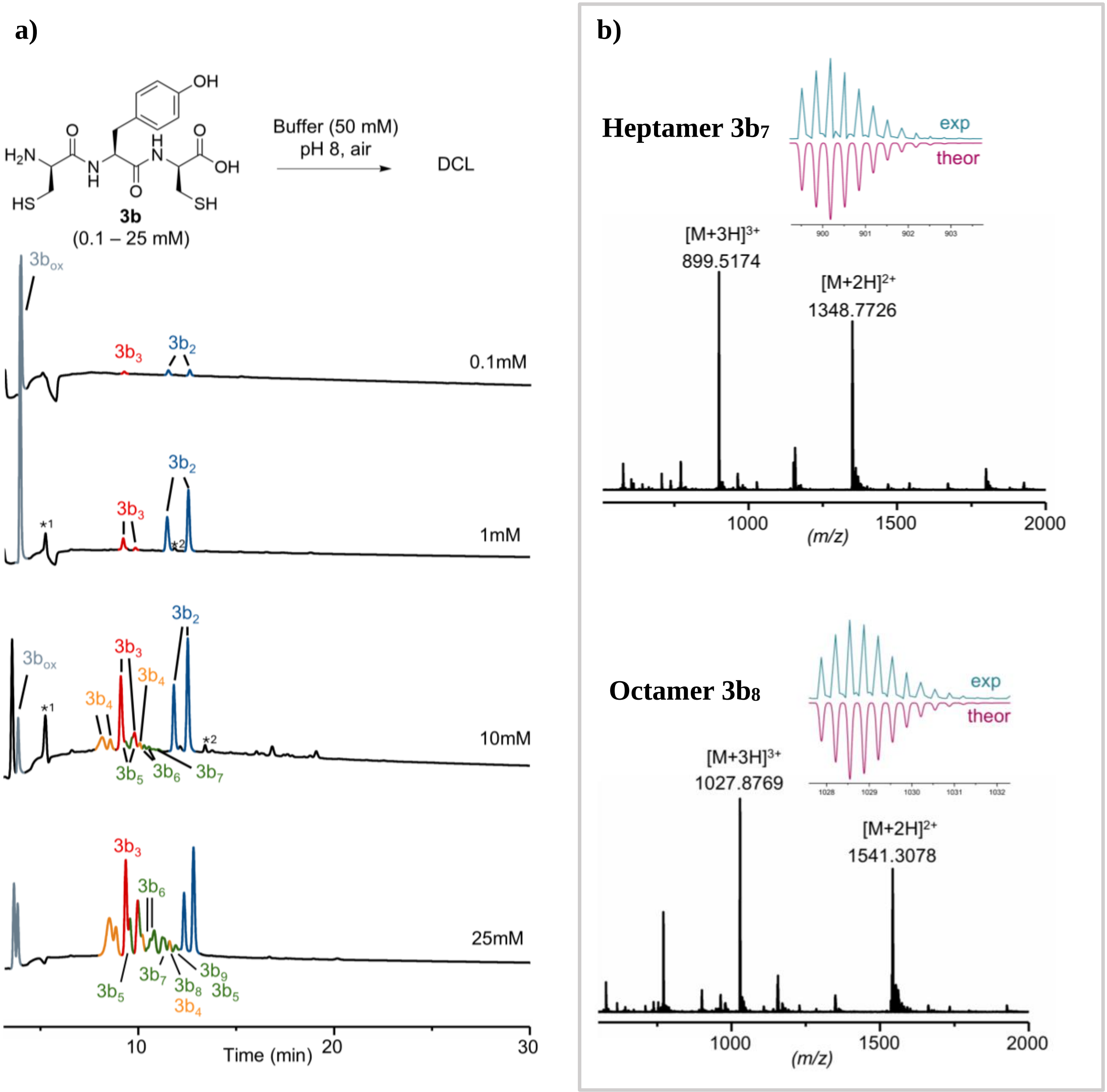

Figure S19. a) UHPLC chromatograms of the libraries generated from peptide **3b** at different concentration. Elution conditions: C18 XSelect peptide CSH, gradient acetonitrile / 0.1 % TFA in H_2O = 15/85 to 40/60 in 30 min (absorbance recorded at 220 nm). b) ESI^+-MS spectra of the corresponding new oxidized products found in the libraries.

5.1.4 Peptide 4b

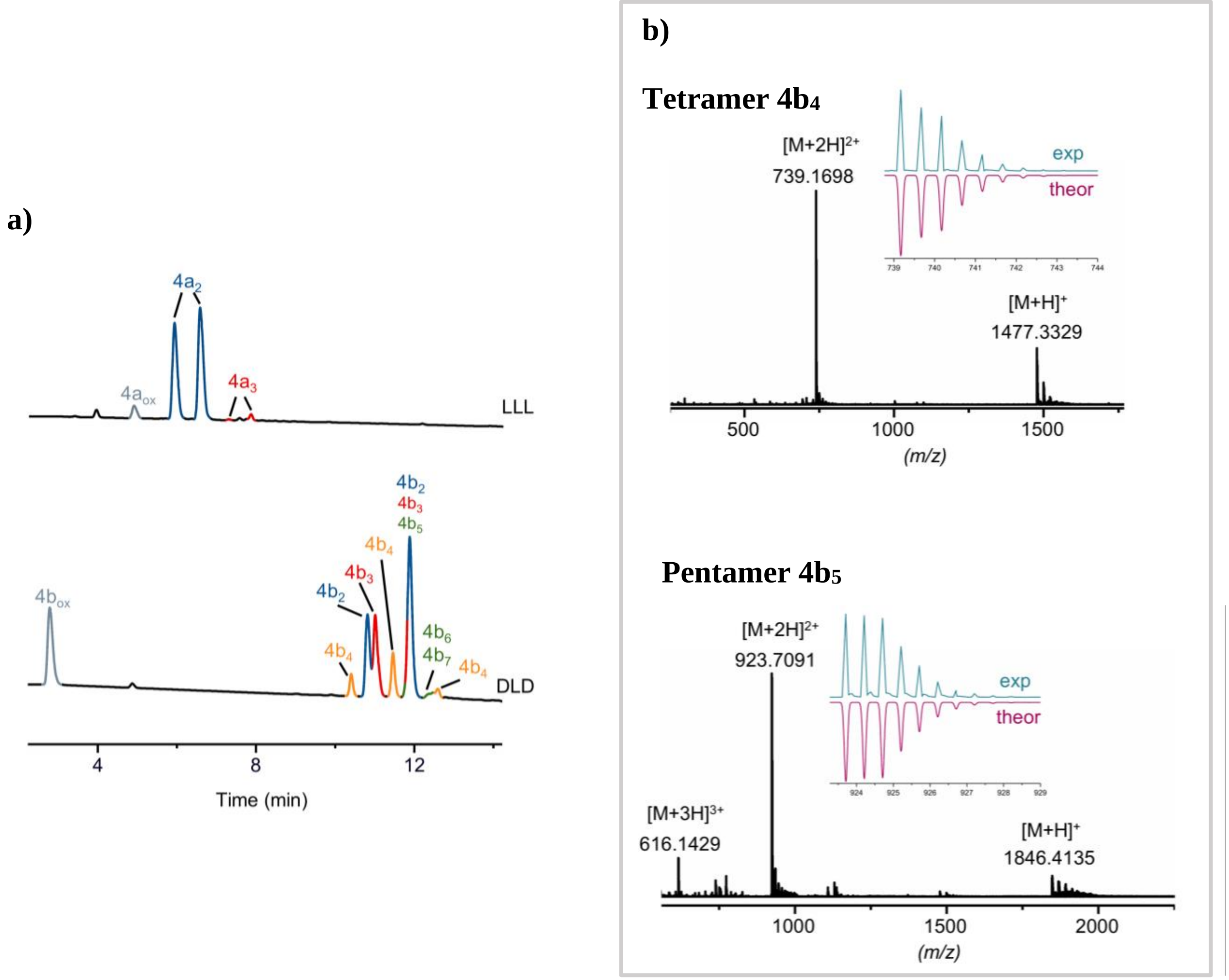

Figure S20. a) UHPLC chromatograms comparison of the libraries generated from the homochiral peptide **4a** and the heterochiral peptide **4b** (10 mM). Elution conditions: C18 XBridge BEH, gradient acetonitrile / 0.1 % TFA in H_2O = 5/95 to 80/20 in 30 min (absorbance recorded at 220 nm). b) ESI^+-MS spectra of the corresponding new oxidized products found in the library. The ESI^+-MS spectra of the higher oligomers are shown on the next page (**Figure S21**).

Hexamer $4b_6$ and Heptamer $4b_7$

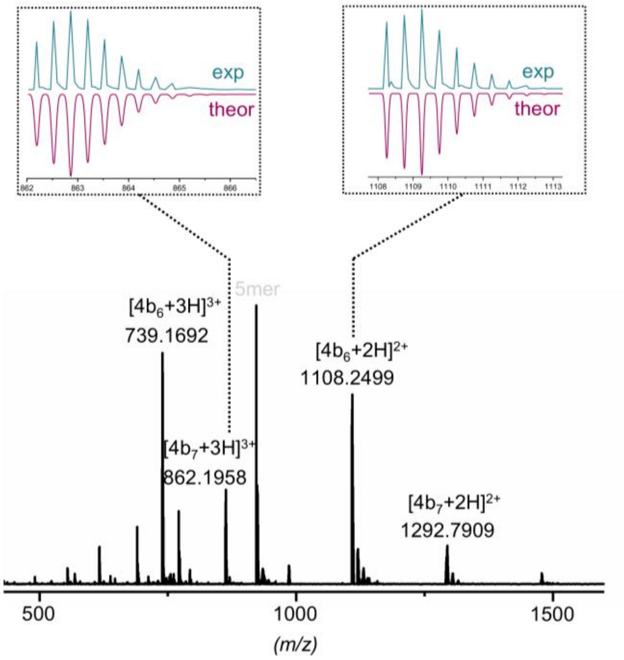

Figure S21. ESI$^+$-MS spectra of hexamer **4b**$_6$ and heptamer **4b**$_7$.

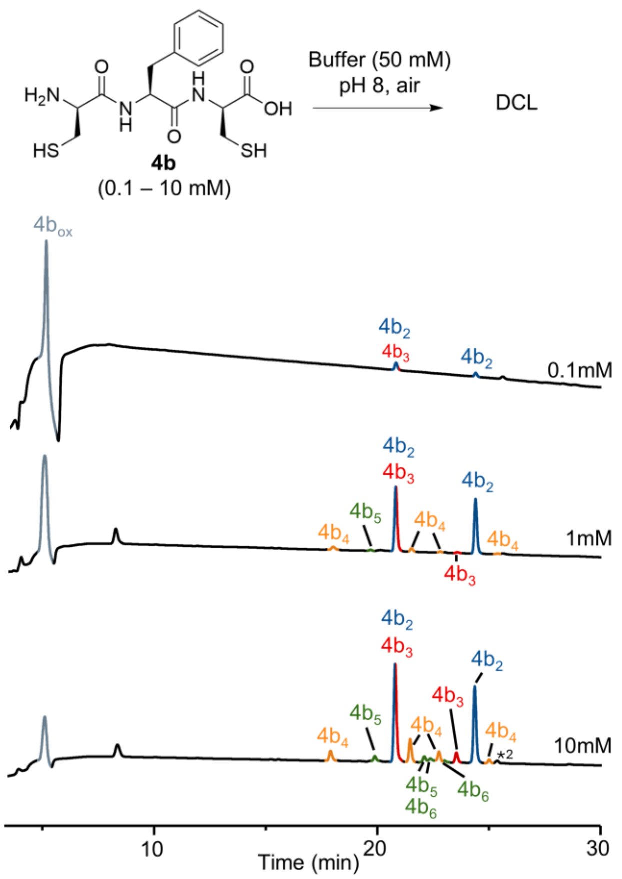

Figure S22. UHPLC chromatograms of the libraries generated from peptide **4b** at different concentration. Elution conditions: C18 XSelect peptide CSH, gradient acetonitrile / 0.1 % TFA in H_2O = 15/85 to 40/60 in 30 min (absorbance recorded at 220 nm). Overoxidized monomer and dimer is labelled with ($*^2$).

5.2 Effect of High salt concentration (1M NaCl)

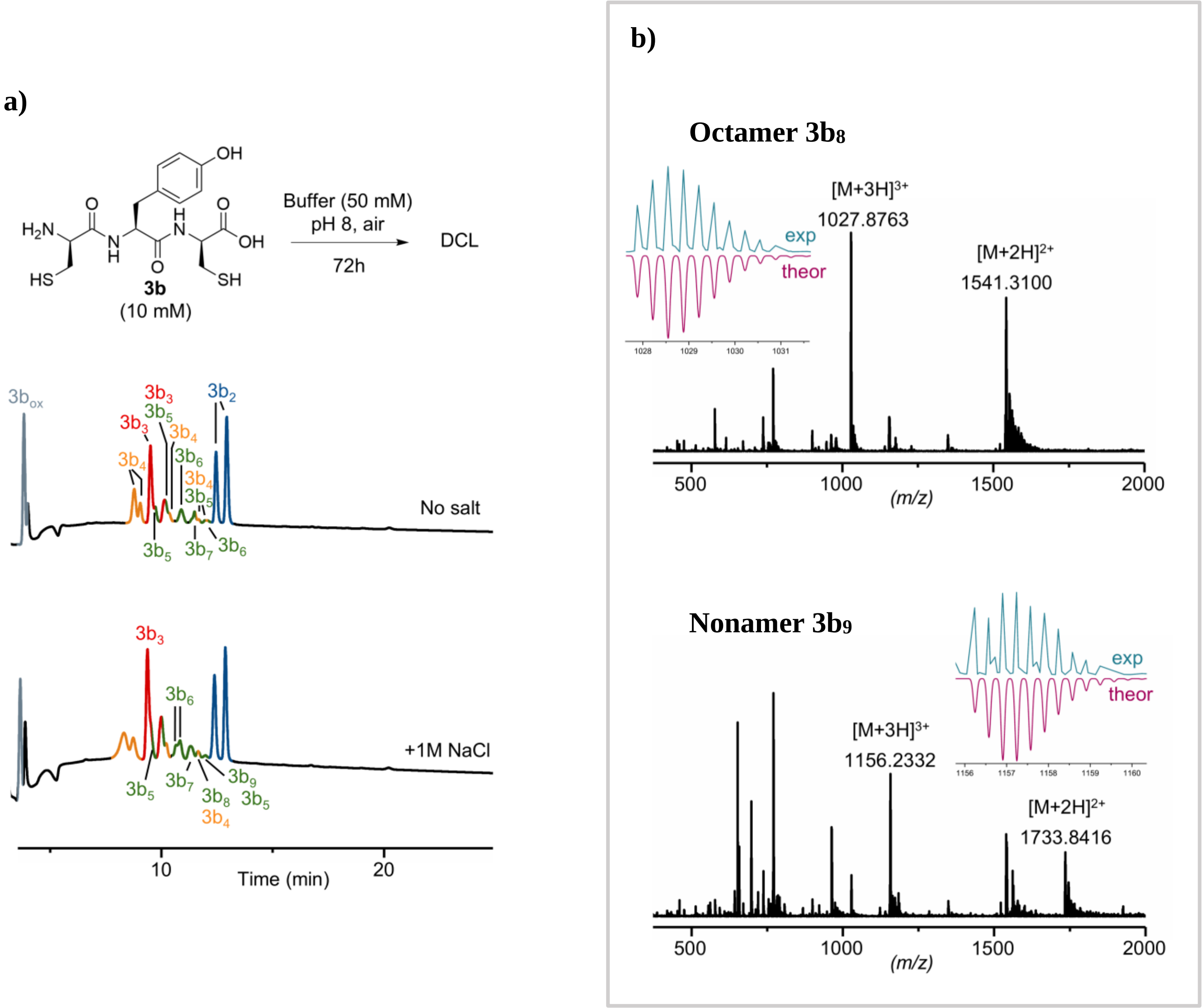

Figure S23. a) UHPLC chromatograms of the libraries generated from peptide **3b** (10 mM) without and with 1M of NaCl at equilibrium. C18 XSelect peptide CSH column, gradient acetonitrile / 0.1 % TFA in H_2O = 15/85 to 40/60 in 30 min (absorbance recorded at 220 nm). b) ESI^+-MS spectra of the corresponding new oxidized products found in the library.

6. Libraries generated from mixture of tripeptides

Representative examples of libraries generated from mixtures of tripeptides (5 mM each) are shown in this section. We limited our study to mixtures of two different tripeptides, as peak overlapping already makes the analysis difficult in most cases.

6.1 Libraries generated from mixture of different homochiral LLL-tripeptides

a)

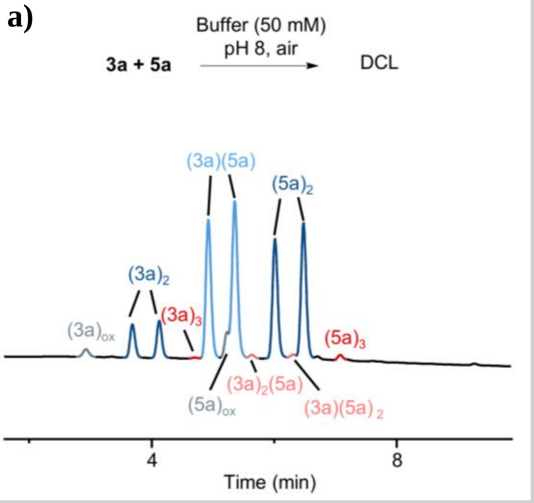

b)

Heterodimer 3a5a

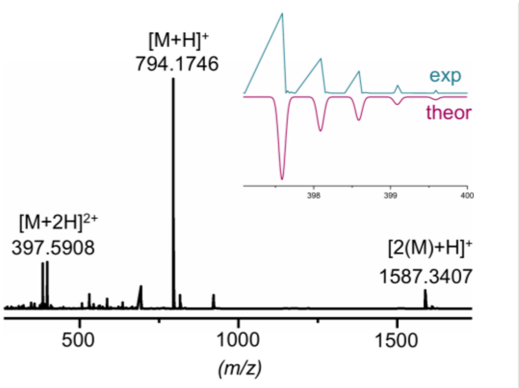

Heterotrimer 3a25a

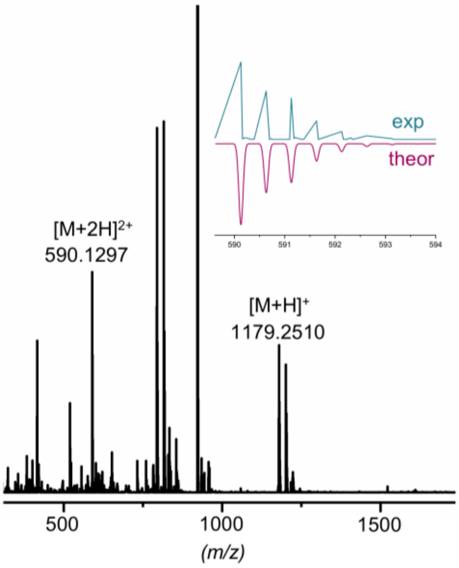

Heterotrimer 3a5a2

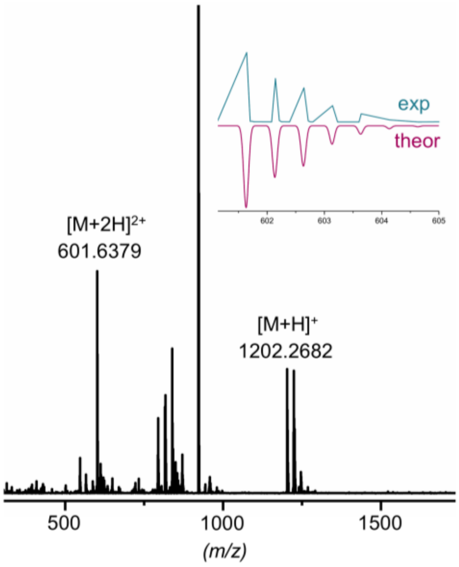

Figure S24. a) UHPLC chromatogram of the libraries generated from mixed peptide **3a** (5mM) and **5a** (5mM). Elution conditions: C18 XBridge BEH, gradient acetonitrile / 0.1 % TFA in H_2O = 5/95 to 80/20 in 20 min (absorbance recorded at 220 nm). b) ESI^+-MS spectra of the corresponding new heteropeptidic oxidized products found in the libraries.

6.2 Libraries generated from mixture of different heterochiral DLD-tripeptides

a)

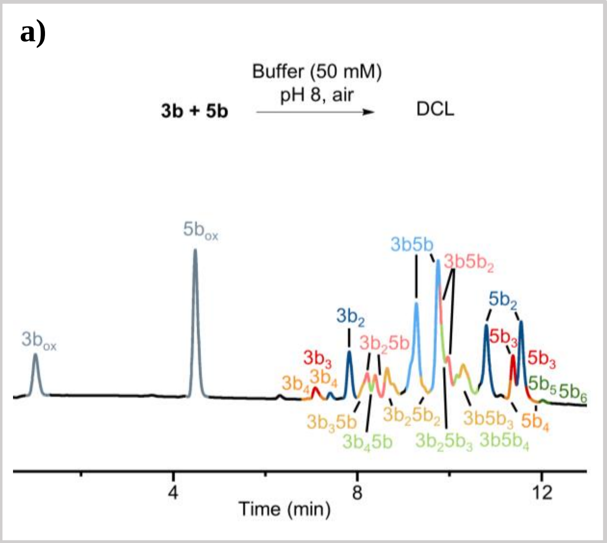

b)

Heterodimer 3b5b

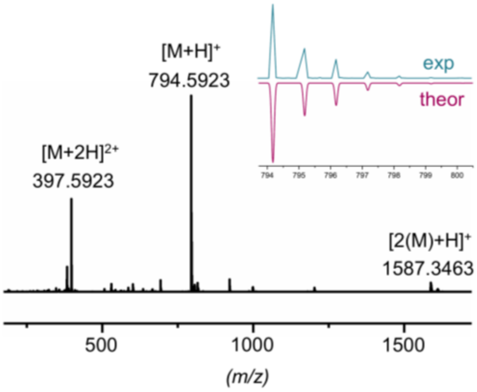

Heterotrimer $3b_25b$

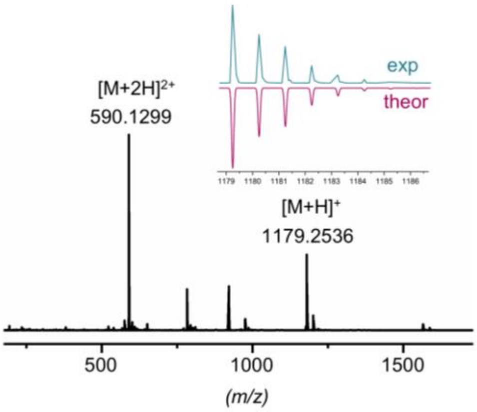

Heterotrimer $3b5b_2$

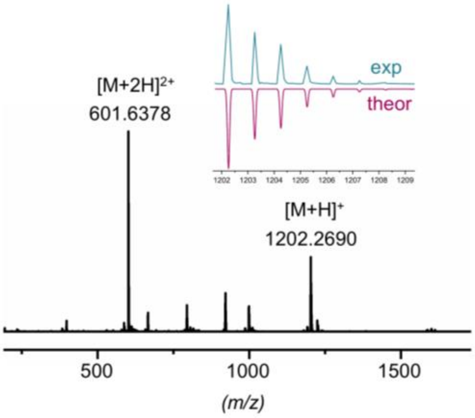

Figure S25. a) UHPLC chromatogram of the libraries generated from mixed peptide **3b** (5 mM) and **5b** (5 mM). Elution conditions: C18 XBridge BEH, gradient acetonitrile / 0.1 % TFA in H_2O = 5/95 to 80/20 in 30 min (absorbance recorded at 220 nm). b) ESI^+-MS spectra of the corresponding new heteropeptidic oxidized products found in the libraries. The ESI^+-MS spectra of the higher oligomers are shown on the next page.

Heterotetramer 3b$_3$5b

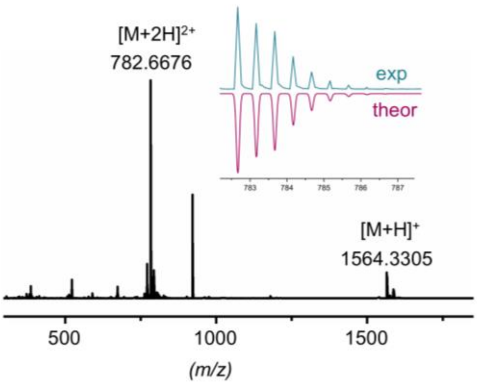

Heterotetramer 3b$_2$5b$_2$

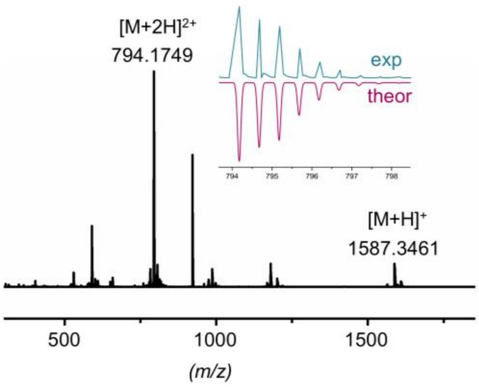

Heterotetramer 3b5b$_3$

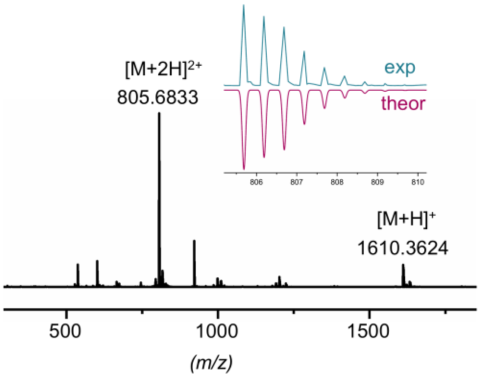

Heteropentamer 3b$_4$5b

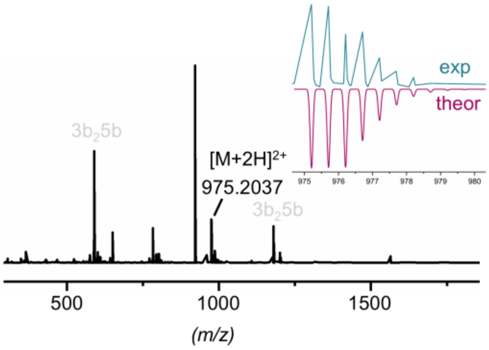

Heteropentamer 3b$_2$5b$_3$

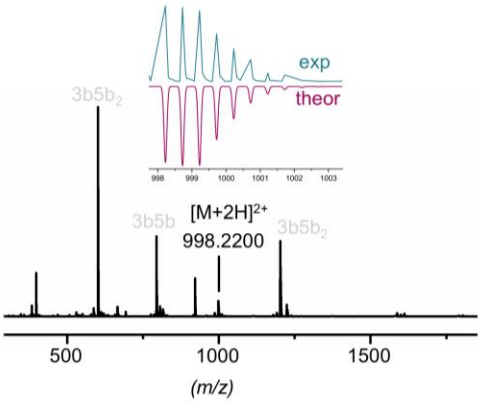

Heteropentamer 3b5b$_4$

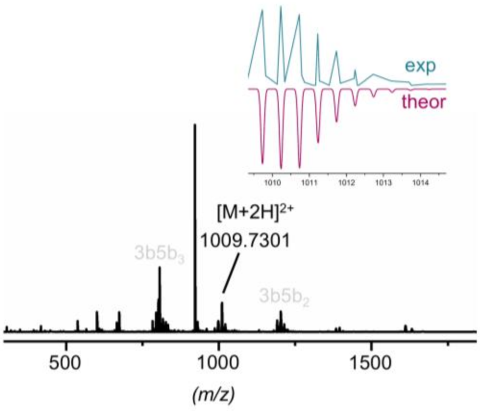

Figure S26. ESI$^+$-MS spectra of heterotetramers and heteropentamers.

6.3 Libraries generated from mixtures of homochiral LLL- and heterochiral DLD-tripeptides analogues

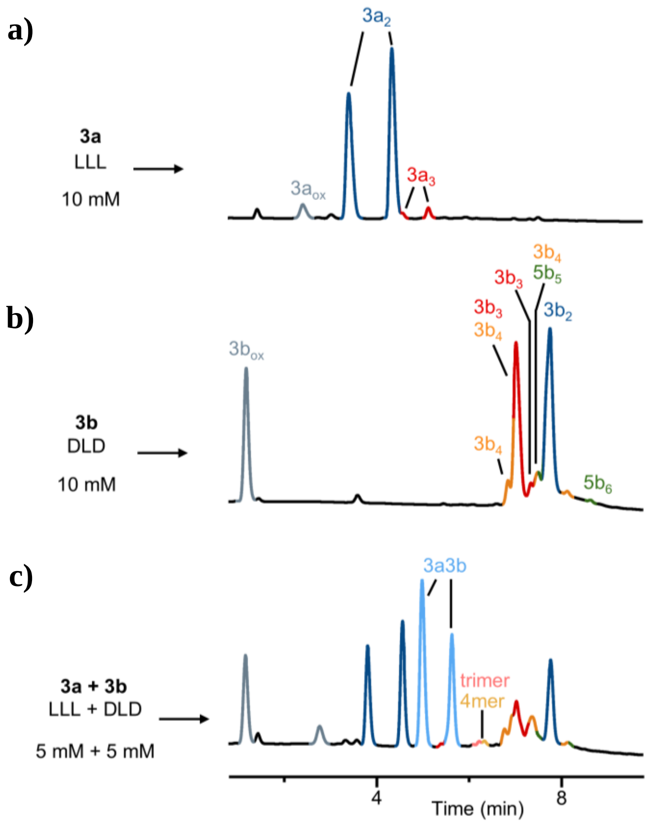

Figure S27. a) UHPLC chromatogram of the libraries generated from homochiral peptide **3a** (10 mM), b) from heterochiral peptide **3b** (10 mM) and c) from mixture of homochiral peptide **3a** and heterochiral peptide **3b** (5 mM each). Elution conditions: C18 XBridge BEH, gradient acetonitrile / 0.1 % TFA in H_2O = 5/95 to 80/20 in 30 min (absorbance recorded at 220 nm).

7. Dynamic combinatorial libraries generated from tripeptides 5a-c

7.1 Concentration-dependent libraries of peptide 5a

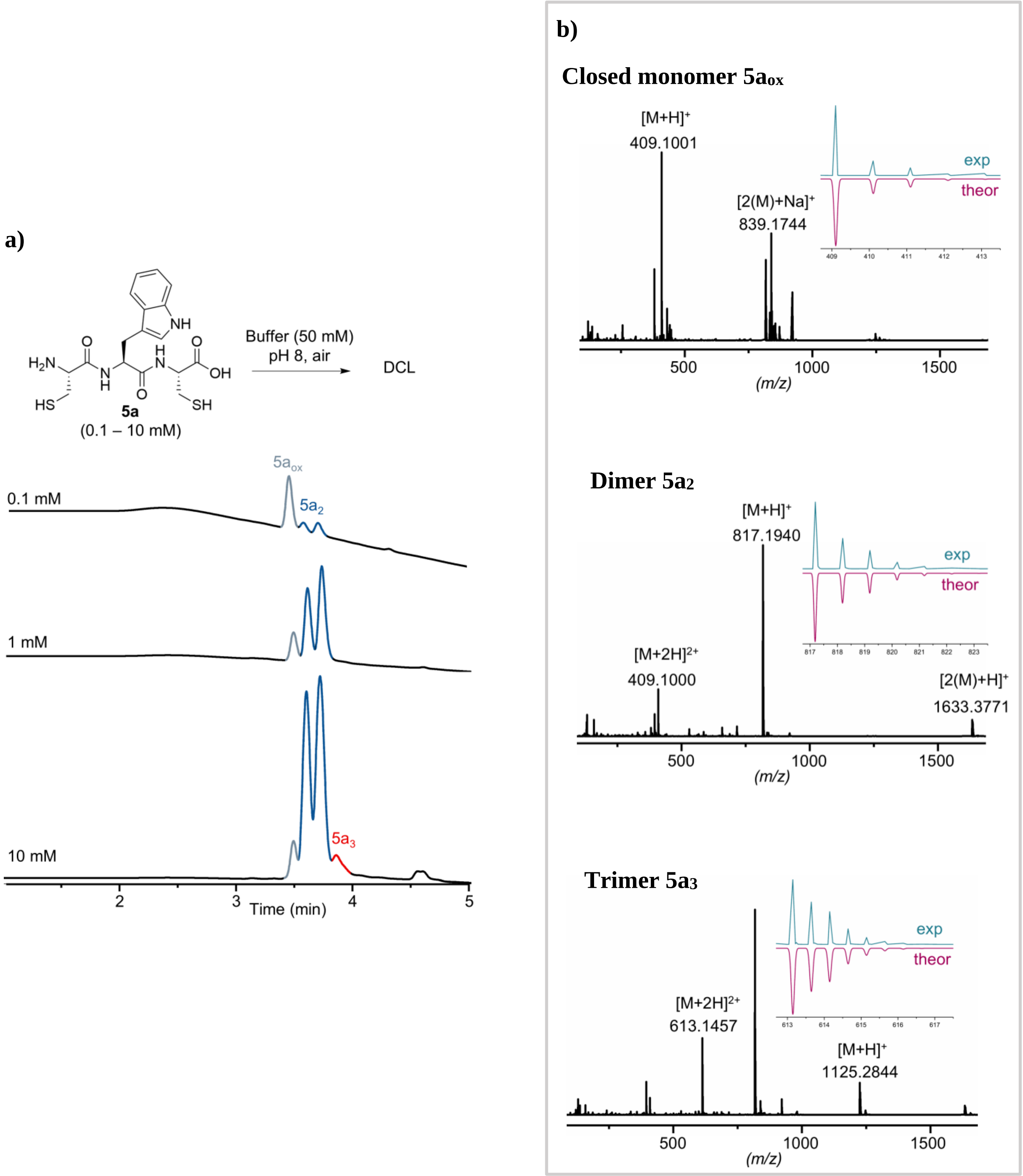

Figure S28. a) UHPLC chromatograms of the libraries generated from peptide **5a** at different concentration. Elution conditions: C18 XBridge BEH, gradient acetonitrile / 0.1 % TFA in H_2O = 5/95 to 80/20 in 6 min (absorbance recorded at 220 nm). b) ESI$^+$-MS spectra of the corresponding oxidized products found in the libraries.

7.2 Libraries generated from peptides 5a and 5b at 10mM

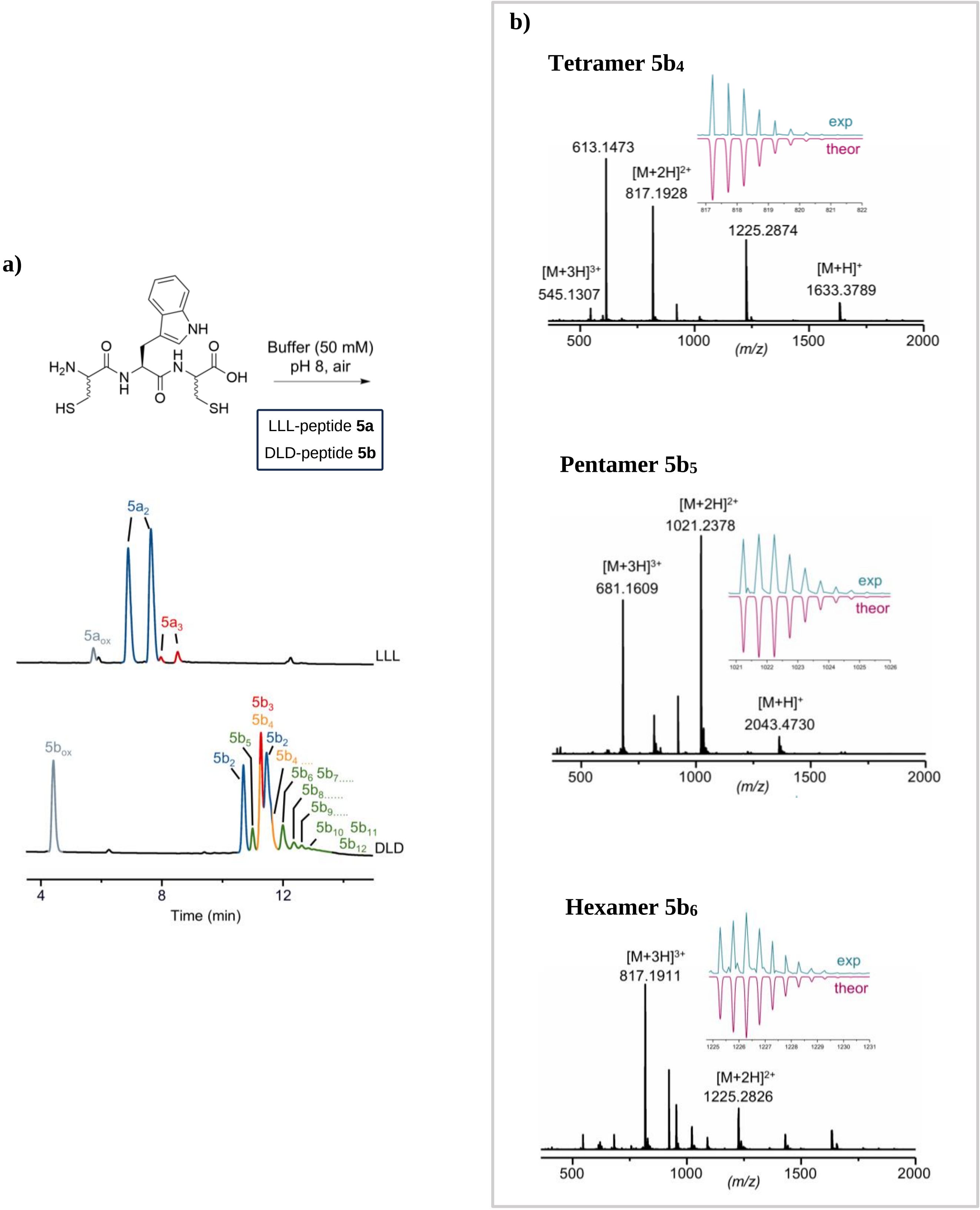

Figure S29. a) UHPLC chromatograms comparison of the libraries generated from the homochiral peptide **5a** and the heterochiral peptide **5b** (10 mM). Elution conditions: C18 XBridge BEH, gradient acetonitrile / 0.1 % TFA in H_2O = 5/95 to 80/20 in 30 min (absorbance recorded at 220 nm). b) ESI$^+$-MS spectra of the corresponding new oxidized products found in the library. The overlapping higher oligomers spectra are shown next page (**Figures S30-S31**).

Heptamer 5b$_7$ and Octamer 5b$_8$

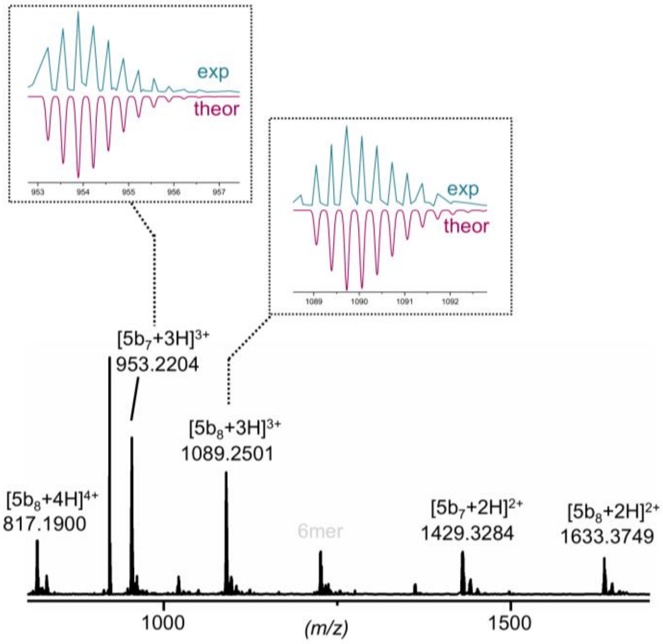

Figure S30. ESI$^+$-MS spectra of the heptamer **5b**$_7$ and octamer **5b**$_8$.

Nonamer 5b$_9$, Decamer 5b$_{10}$ and Undecamer 5b$_{11}$

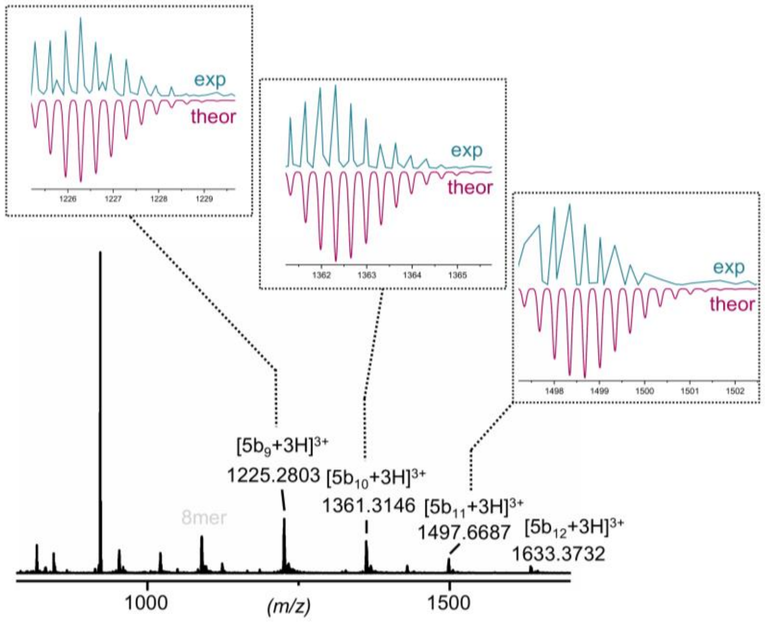

Figure S31. ESI$^+$-MS spectra of nonamer **5b**$_9$, decamer **5b**$_{10}$ and undecamer **5b**$_{11}$. Traces of dodecamer **5b**$_{12}$ were also observed.

7.3 Concentration-dependent libraries of peptide 5b

a)

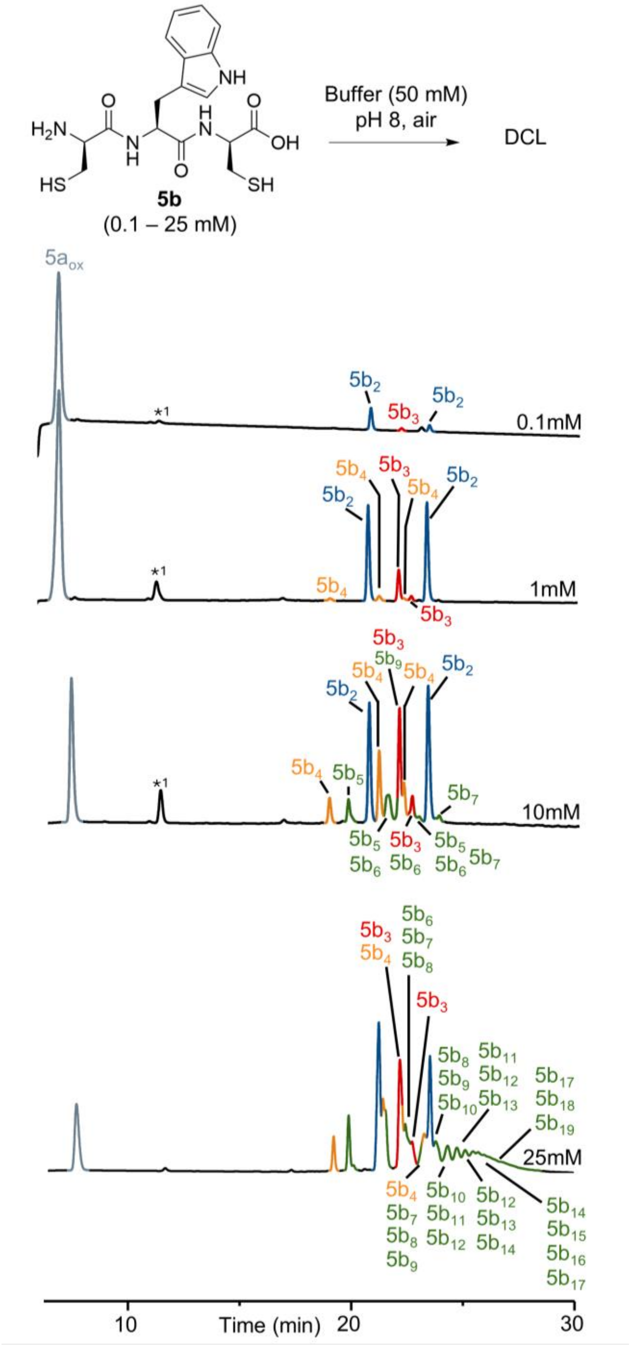

b)

Tridecamer 5b13

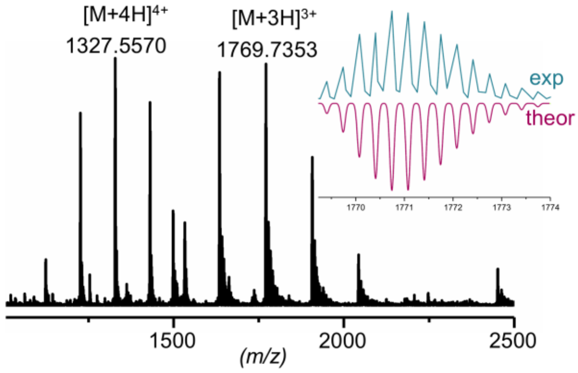

Hexadecamer 5b16

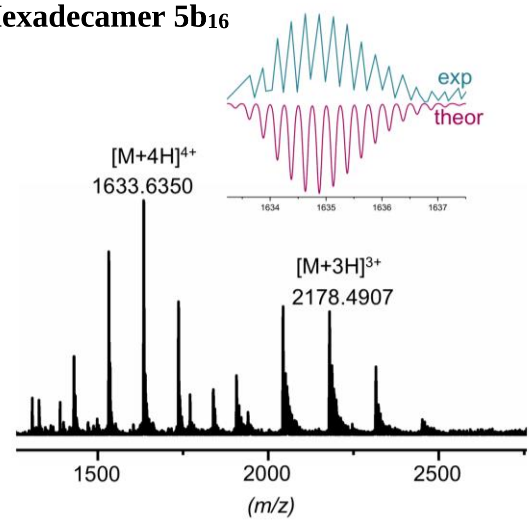

Figure S32. a) UHPLC chromatograms of the libraries generated from peptide **5b** at different concentration. Elution conditions: C18 XSelect peptide CSH, gradient acetonitrile / 0.1 % TFA in H_2O = 15/85 to 40/60 in 30 min (absorbance recorded at 220 nm). Overoxidized monomer is labelled with (*1). b) ESI+-MS spectra of the corresponding new oxidized products found in the library. ESI+-MS spectra of the other oligomers are shown on the next page (**Figures S33-S34**).

Tetradecamer 5b14 and Pentadecamer 5b15

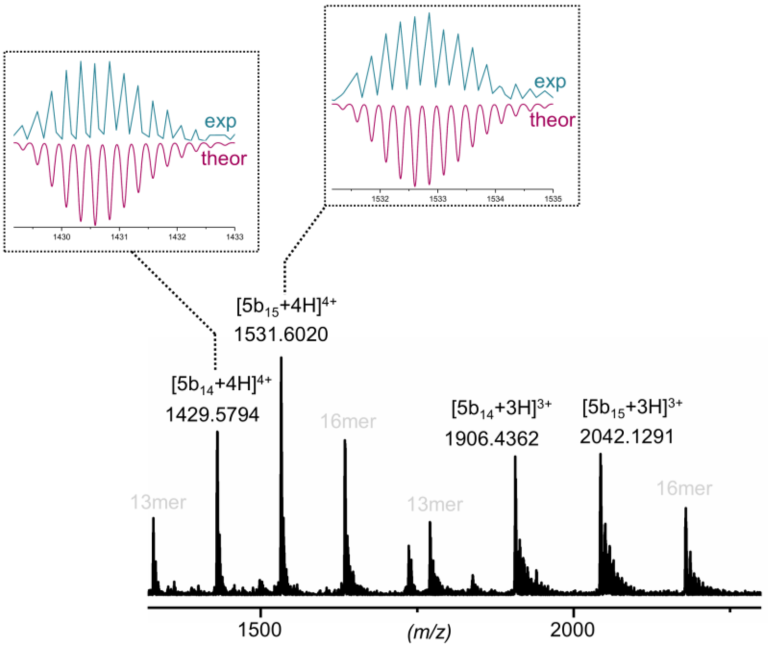

Figure S33. ESI$^+$-MS spectra of the tetradecamer **5b**$_{14}$ and pentadecamer **5b**$_{15}$

Heptadecamer 5b17 and Octadecamer 5b18

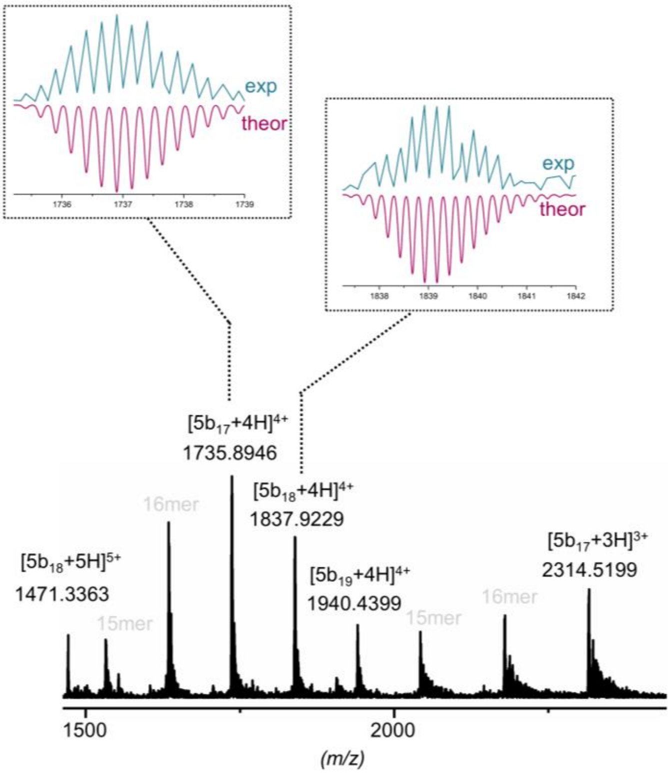

Figure S34. ESI$^+$-MS spectra of heptadecamer **5b**$_{17}$ and octadecamer **5b**$_{18}$, and nonadecamer **5b**$_{19}$.

7.4 Concentration-dependent library of peptide 5c

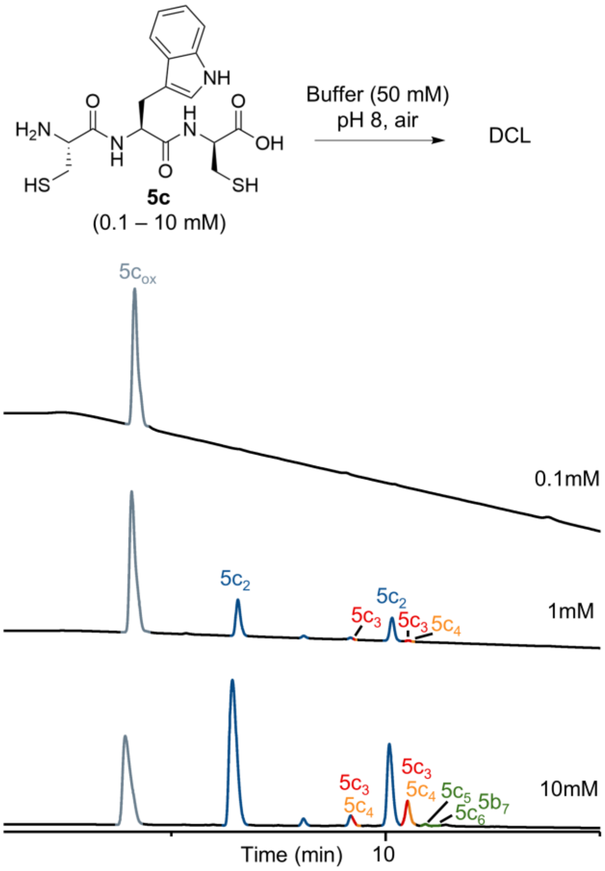

Figure S35. UHPLC chromatograms of the libraries generated from peptide **5c** at different concentration. Elution conditions: C18 XBridge BEH, gradient acetonitrile / 0.1 % TFA in H_2O = 5/95 to 80/20 in 30 min (absorbance recorded at 220 nm).

7.5 CD spectra of isolated monomers and dimers from DCLs generated by peptides 5a-c

7.5.1 Monomer and dimers of peptide 5a

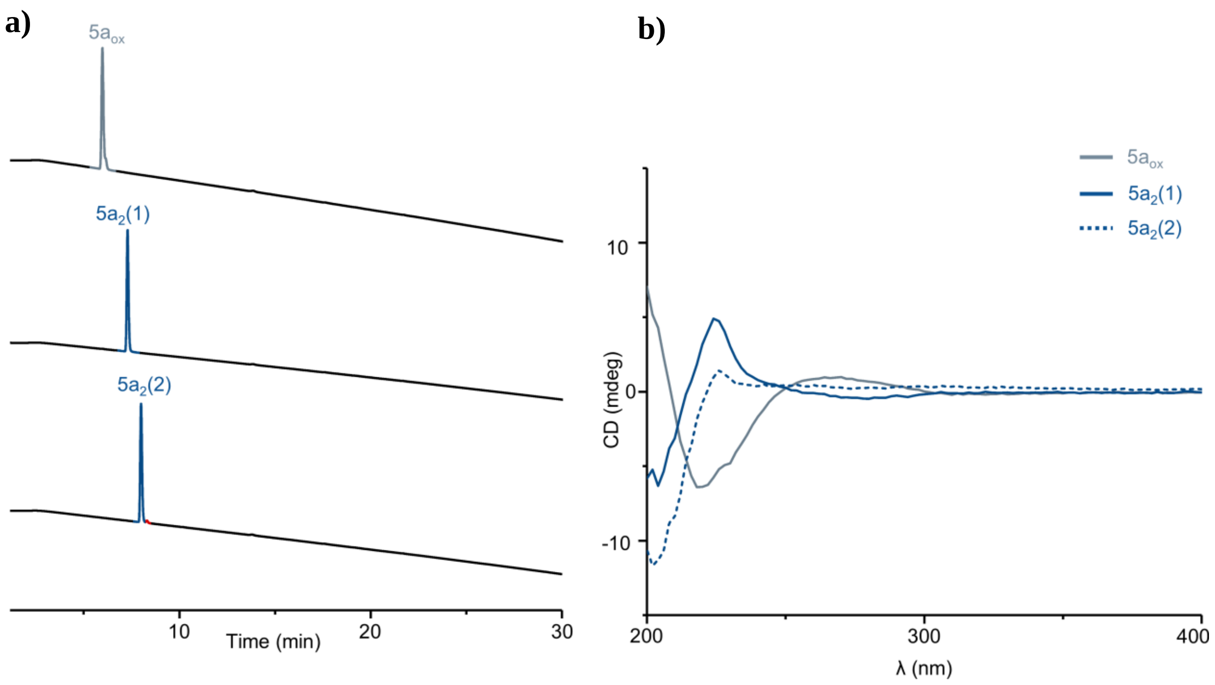

Figure S36. a) UHPLC chromatograms of isolated closed monomer **5a**$_{ox}$, first eluting dimer **5a**$_2$(1) and second eluting dimer **5a**$_2$(2). Elution conditions: C18 XBridge BEH, gradient acetonitrile / 0.1 % TFA in H_2O = 5/95 to 80/20 in 30 min (absorbance recorded at 220 nm). The peak corresponding to a residual trace of trimer in the isolated sample of 5a$_2$(2) is colored in red. b) corresponding CD spectra recorded at 0.08 mM for the monomer and 0.04 mM for the dimers in a 5 mm cuvette.

7.5.2 Monomer and dimers of peptide 5b

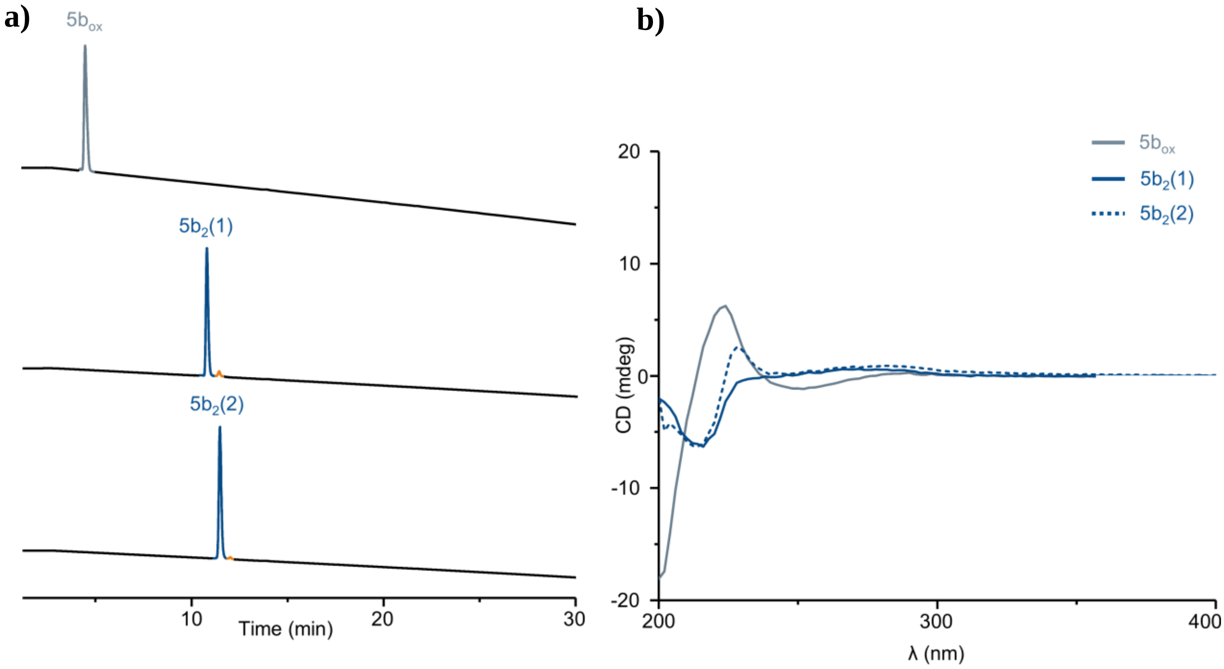

Figure S37. a) UHPLC chromatograms of isolated closed monomer **5b**$_{ox}$, first eluting dimer **5b**$_2$(1) and second eluting dimer **5b**$_2$(2), Elution conditions: C18 XBridge BEH, gradient acetonitrile / 0.1 % TFA in H_2O = 5/95 to 80/20 in 30 min (absorbance recorded at 220 nm). The peaks corresponding to a residual trace of tetramer in the samples of isolated 5b$_2$(1) and 5b$_2$(2) are colored in orange. b) corresponding CD spectra recorded at 0.08 mM for the monomer and 0.04 mM for the dimers in a 5 mm cuvette.

7.5.3 Monomer and dimers of peptide 5c

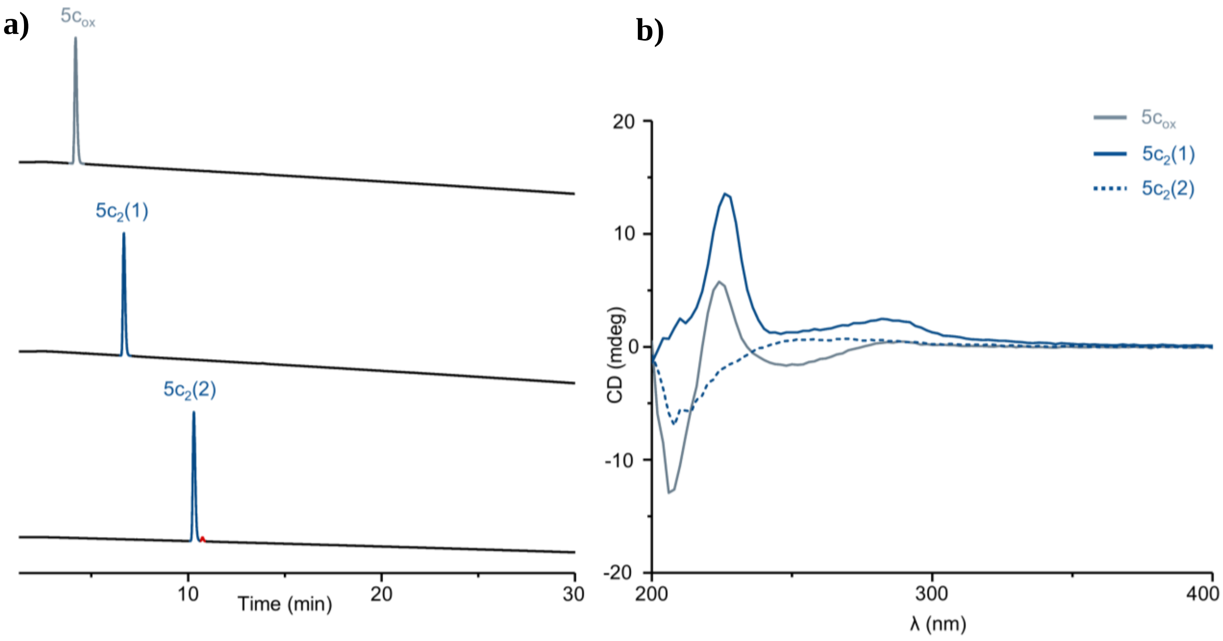

Figure S38. a) UHPLC chromatograms of isolated closed monomer **5c**$_{ox}$, first eluting dimer **5c**$_2$(1) and second eluting dimer **5c**$_2$(2). Elution conditions: C18 XBridge BEH, gradient acetonitrile / 0.1 % TFA in H_2O = 5/95 to 80/20 in 30 min (absorbance recorded at 220 nm). The peak corresponding to a residual trace of trimer in the sample of isolated 5c$_2$(2) is colored in red. b) corresponding CD spectra recorded at 0.08 mM for the monomer and 0.04 mM for the dimers in a 5 mm cuvette.

7.6 IM-MS of isolated monomers and dimers from DCLs generated by peptides 5a-c

	Monomer		Dimer 1		Dimer 2	
	Drift time (ms)	CCS (Å^2)	Drift time (ms)	CCS (Å^2)	Drift time (ms)	CCS (Å^2)
Peptide 5a (LLL)	25.10	188.8	35.16	261.4	35.78	267.7
Peptide 5b (DLD)	25.38	190.7	35.23	261.7	35.00	259.4
Peptide 5c (LLD)	24.72	186.3	34.91	259.9	36.09	268.8

Table S2. IM-MS results showing drift time and CCS value for all isolated monomers, first eluting dimers (1) and second eluting dimers (2) of peptides **5a**, **5b** and **5c**.

7.7 MD simulations of dimers 5a2 and 5b2

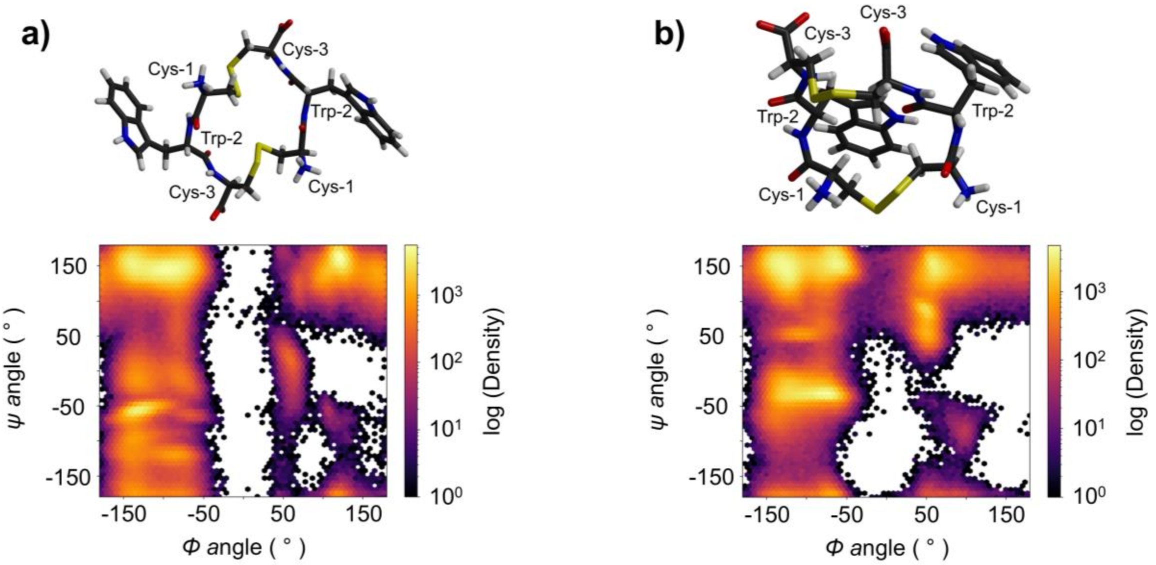

Figure S39. Representative structure and Ramachandran plots, obtained from cluster analysis of MD trajectories, of the anti-parallel (a) and parallel (b) dimers **5a**2.

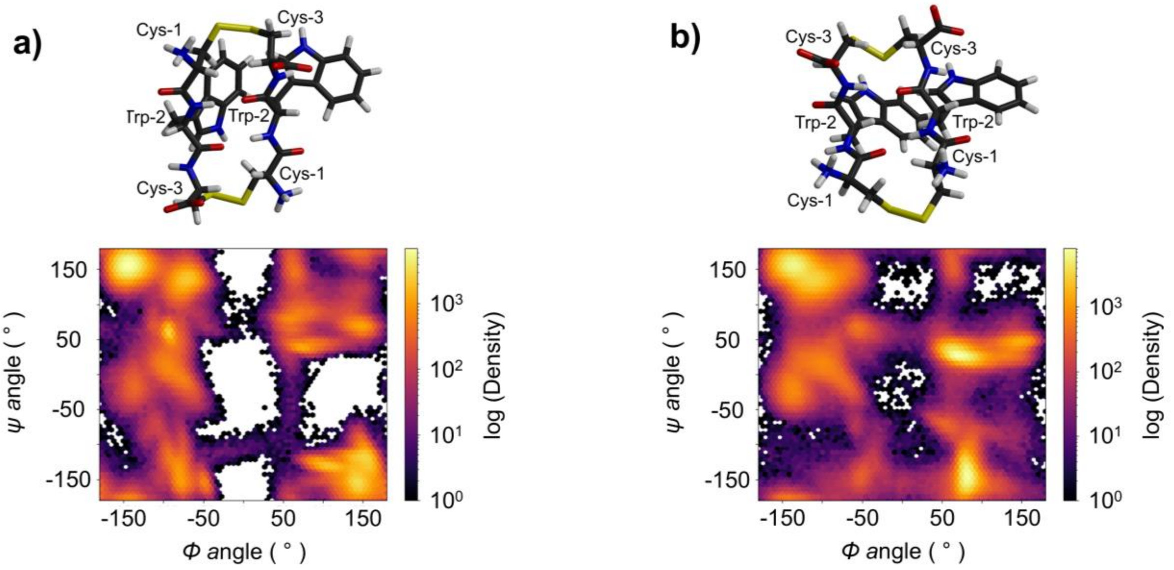

Figure S40. Representative structure and Ramachandran plots, obtained from cluster analysis of MD trajectories, of the anti-parallel (a) and parallel (b) dimers **5b**2.

8. Libraries generated from tetrapeptides

a)

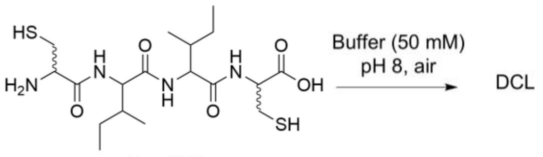

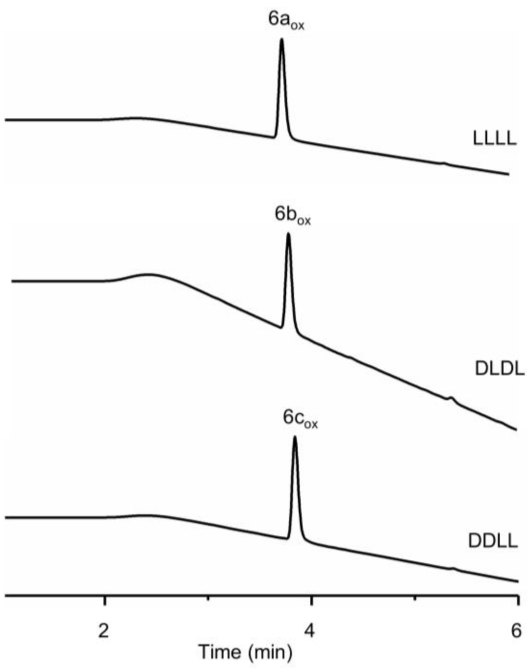

b) Closed monomer 6b$_{ox}$

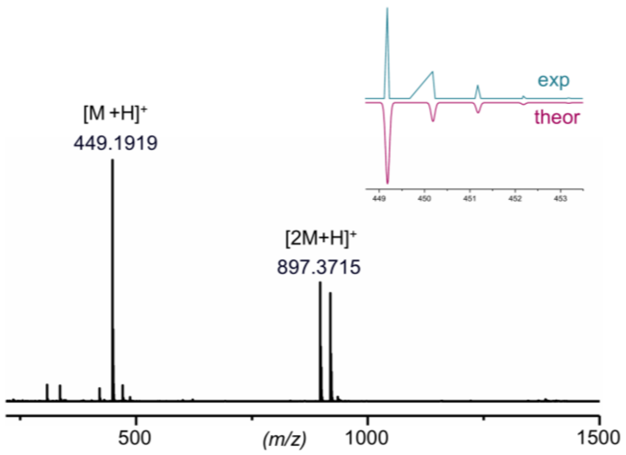

Figure S41. a) UHPLC chromatogram of the libraries generated from peptide **6a**, **6b** and **6c** (1 mM). Elution conditions: C18 XBridge BEH, gradient acetonitrile / 0.1 % TFA in H_2O = 5/95 to 80/20 in 6 min (absorbance recorded at 220 nm). b) corresponding ESI$^+$-MS spectrum.

9. ^{1}H NMR spectra of the new peptides

9.1 Peptides 1a and 1b

a)

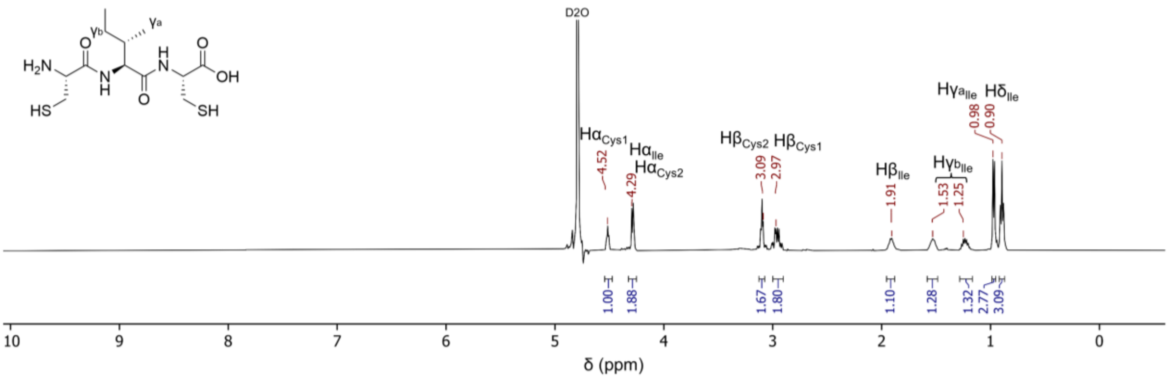

b)

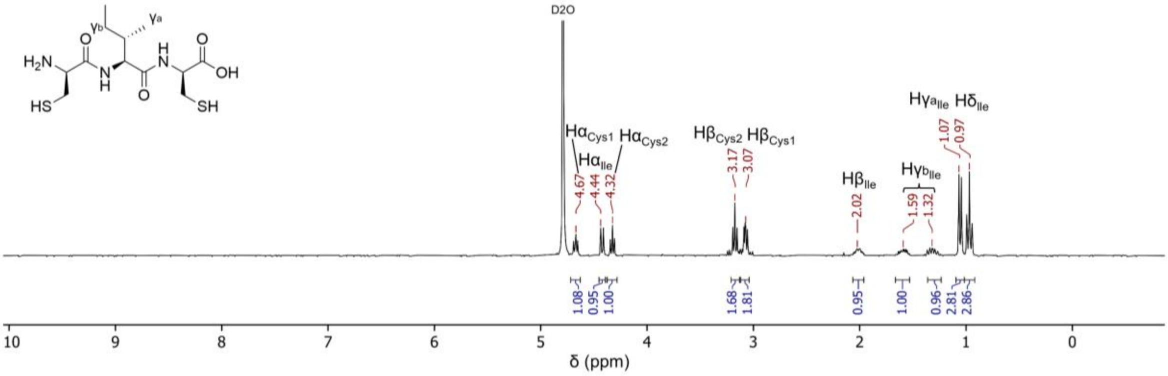

Figure S42. ^{1}H NMR spectra of a) peptide **1a** (500 MHz, D_2O, 25°C) and b) peptide **1b** (300 MHz, D_2O, 30°C).

9.2 Peptides 2a and 2b

a)

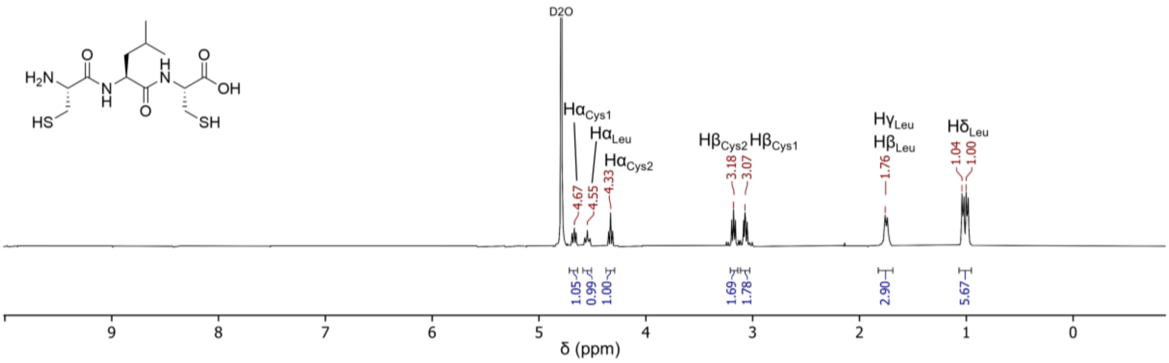

b)

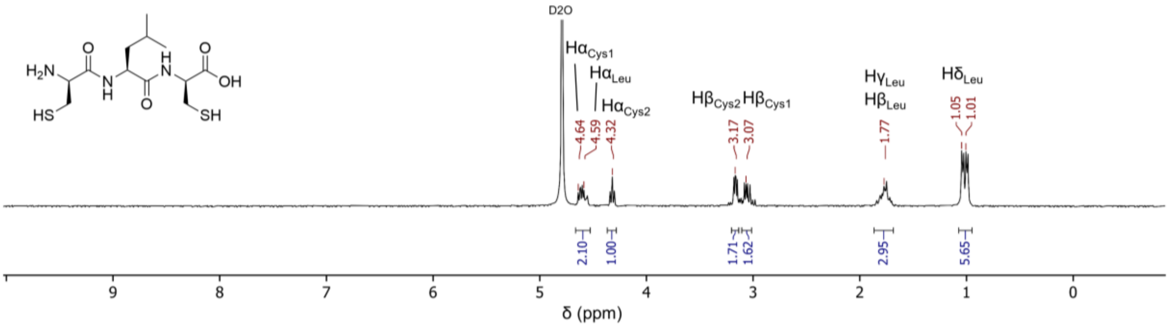

Figure S43. ^{1}H NMR spectra of a) peptide **2a** and b) peptide **2b** (300 MHz, D_2O, 30°C).

9.3 Peptide 3b

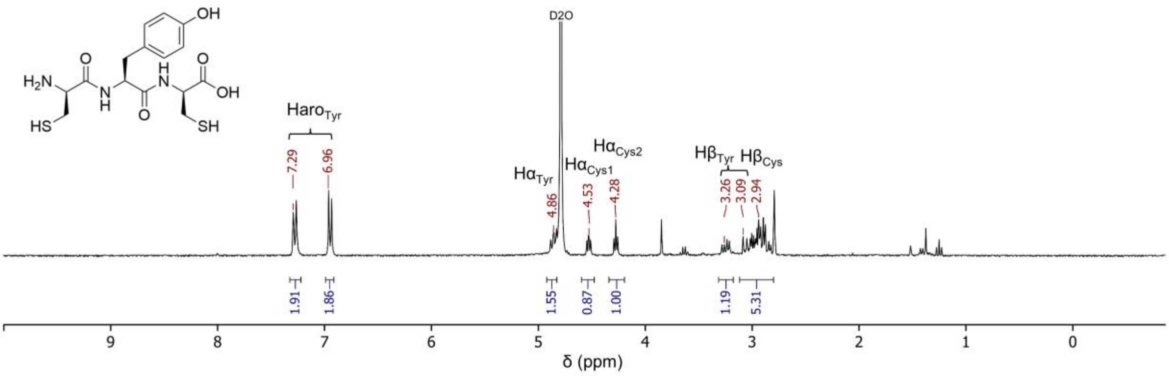

Figure S44. ^{1}H NMR spectrum of peptide **3b** (300 MHz, D_2O, 30°C).

9.4 Peptide 4b

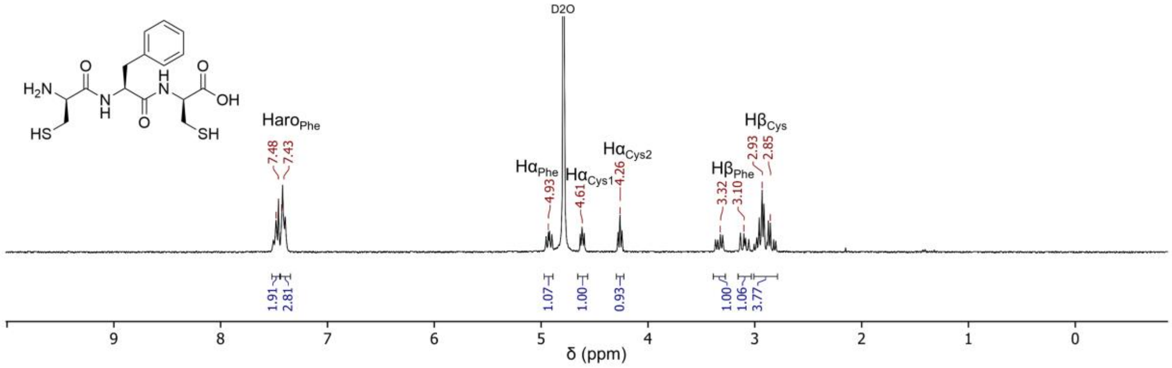

Figure S45. ^{1}H NMR spectrum of peptide **4b** (300 MHz, D_2O, 30°C).

9.5 Peptides 5b and 5c

a)

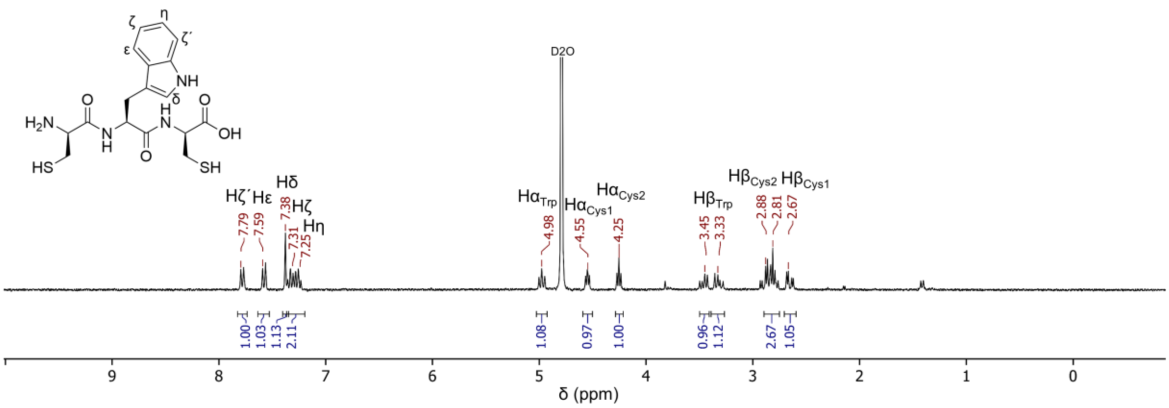

b)

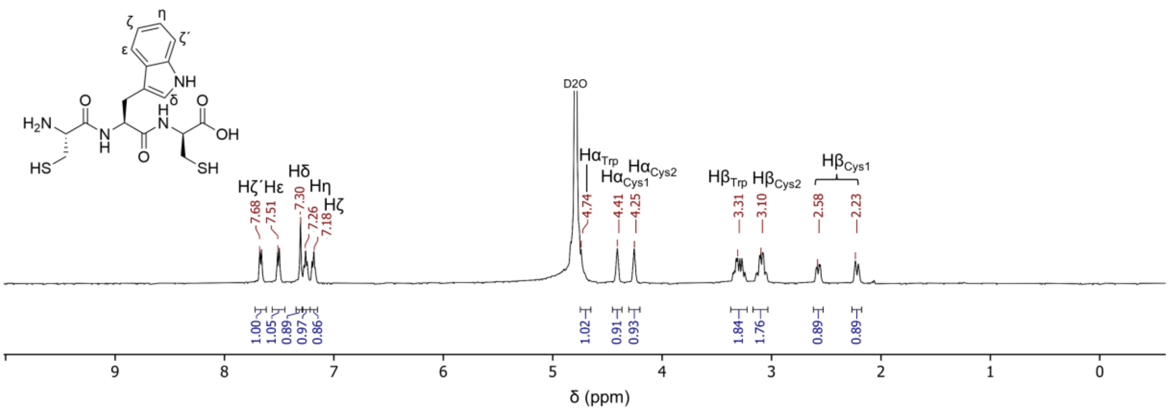

Figure S46. ^{1}H NMR spectra of a) peptide **5a** (300 MHz, D_2O, 30°C) and b) peptide **5b** (500 MHz, D_2O, 25°C).

9.6 Peptides 6a, 6b and 6c

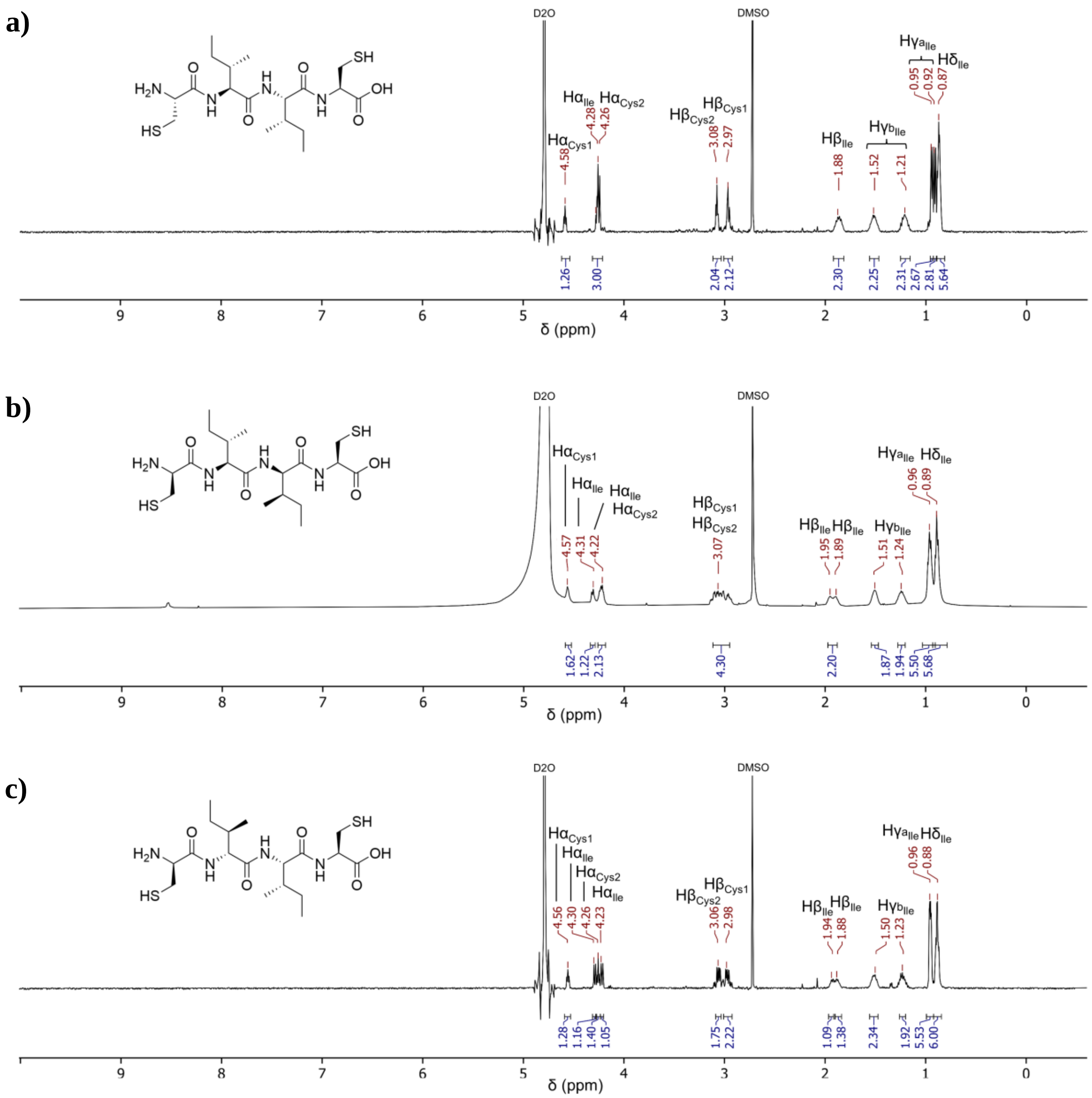

Figure S47. ^{1}H NMR spectra of a) peptide **6a,** b) peptide **6b** and c) peptide **6c** (500 MHz, D_2O, 25°C). DMSO was added to the samples due to solubility issues.

10. References

[1] M. J. Abraham, T. Murtola, R. Shulz, S. Páll, J. C. Smith, B. Hess, E. Lindahl, "GROMACS: High performance molecular simulations through multi-level parallelism from laptops to supercomputers," *SoftwareX*. **2015**, 1-2, 19–25.

[2] D. Van Der Spoel, E. Lindahl, B. Hess, G. Groenhof, A. E. Mark, H. J. C. Berendsen, "GROMACS: Fast, flexible, and free," *J. Comput. Chem.* **2005**, 26, 1701–1718.

[3] K. Lindorff-Larsen, S. Piana, K. Palmo, P. Maragakis, J. L. Klepeis, R. O. Dror, D. E. Shaw, "Improved side-chain torsion potentials for the Amber ff99SB protein force field," *Proteins Struct. Funct. Bioinforma.* **2010**, 78, 1950–1958.

[4] W. L. Jorgensen, J. Chandrasekhar, J. D. Madura, R. W. Impey, M. L. Klein, "Comparison of simple potential functions for simulating liquid water," *J. Chem. Phys.* **1983**, 79, 926–935.

[5] G. Bussi, D. Donadio, M. Parrinello, "Canonical sampling through velocity rescaling," *J. Chem. Phys.* **2007**, 126, 014101.

[6] M. Bernetti, G. Bussi, "Pressure control using stochastic cell rescaling," *J. Chem. Phys.* **2020**, 153, 114107.

[7] B. Hess, H. Bekker, H. J. C. Berendsen, J. G. E. M. Fraaije, "LINCS: A linear constraint solver for molecular simulations," *J. Comput. Chem.* **1997**, 18, 1463–1472.

[8] T. Darden, D. York, L. Pedersen, "Particle mesh Ewald: An N·log(N) method for Ewald sums in large systems," *J. Chem. Phys.* **1993**, 98, 10089–10092

[9] I. Coin, M. Beyermann, M. Bienert, "Solid-phase peptide synthesis: from standard procedures to the synthesis of difficult sequences," *Nat. Protoc.* **2007**, 2, 3247-3256.

[10] A. G. Kreutzer, P. J. Salveson, H. Yang, and G. Guaglianone, "Standard practices for Fmoc-based solid-phase peptide synthesis in the Nowick laboratory (Version 1.7.2)", can be found under https://www.chem.uci.edu/~jsnowick/groupweb/files/Standard_practices_for_Fmoc_based_solid_phase_peptide_synthesis_in_the_Nowick_Laboratory_V_1.7.2.pdf, (accessed: (2025))

[11] "Aapptec Technical Support Information Bulletin 1198.", can be found under https://www.peptide.com/custdocs/1198.pdf, (accessed: (2025)).

[12] M. Rauschenberg, S. Bomke, U. Karst, B. J. Ravoo, "Dynamic Peptides as Biomimetic Carbohydrate Receptors," *Angew. Chem. Int. Ed.* **2010**, 49, 7340–7345.
